# Perioperative Nursing Shortages: An Integrative Review of Their Impact, Causal Factors, and Mitigation Strategies

**DOI:** 10.1155/2024/2983251

**Published:** 2024-08-19

**Authors:** Ada Xie, Jed Duff, Judy Munday

**Affiliations:** ^1^ School of Nursing Queensland University of Technology, Brisbane, QLD, Australia; ^2^ School of Nursing Centre for Healthcare Transformation Queensland University of Technology, Brisbane, QLD, Australia; ^3^ Royal Brisbane and Women's Hospital Centre for Nursing and Midwifery Research, Herston, QLD, Australia; ^4^ School of Health University of the Sunshine Coast, Sunshine Coast, QLD, Australia

## Abstract

**Aim:**

This study aims to explore contributing factors, impacts, and strategies to address perioperative nursing shortages.

**Background:**

Health facilities worldwide are experiencing nursing shortages, especially in specialty fields such as perioperative nursing. *Evaluation*. This integrative review is reported according to the PRISMA guidelines. The title, abstract, and full article screening, as well as the quality appraisal process, were performed by two independent reviewers, with a third for disagreement. This review focused on empirical and theoretical research published from 2013 to 2023 using databases including CINAHL, Embase, Emcare (via OVID), Medline (via EBSCOhost), Scopus, Web of Science, ProQuest Dissertations and Theses Global, Overton, and GreyNet. *Key Issues*. This study thoroughly reviewed 84 articles. The perioperative domain confronts significant staffing challenges due to increased demand, lack of experienced nurses, insufficient new entrants, high turnover, and an aging workforce. Notably significant are the deficiencies in recruiting new nurses and the elevated turnover levels, potentially amendable issues. The shortages negatively impact the remaining nurses, patient care quality, and hospital revenue. Strategies to address perioperative nursing workforce challenges include promoting the specialty to undergraduate nursing students, bolstering recruitment efforts, and retaining experienced perioperative nurses. However, none of the studies examined in this review adopted a comprehensive approach. Furthermore, the effectiveness of these strategies relative to one another remains uncertain due to a lack of reliable measurements.

**Conclusion:**

Perioperative nursing faces considerable challenges, including an aging workforce, limited new recruits, and high turnover rates. Present strategies primarily prioritise workforce preparation over supporting current staff. Mitigating the perioperative nursing shortages requires comprehensive approaches integrating preparation, recruitment, retention, and retirement plans. In addition, these strategies must be adapted to the diverse regulatory environments of different countries, recognising the absence of a one-size-fits-all solution to perioperative nursing shortages globally. *Implication for Nursing Management*. Perioperative nursing managers are vital in reducing shortages.

## 1. Background

Nursing shortages are a severe global problem [1–4]. Worldwide, the World Health Organisation (WHO) report estimated a shortage of 5.7 million nurses by 2030 [5]. The shortage is most acute in the poorest nations, particularly in sub-Saharan Africa, where the shortage is exacerbated by substantial migration from poorer to wealthier countries, driven by salary gaps and recruitment initiatives in developed nations [6, 7]. High nurse turnover and problems with retention compound nursing workforce shortages [2, 8–13]. In the United Kingdom (UK), the Nursing and Midwifery Council reported that 27% more nurses are leaving the profession than joining [2]. Worldwide, studies report turnover rates from 12% to 44.3% [10, 13, 14] and intention-to-leave rates from 42.9% to 94% [15–17]. An aging population also contributes to nursing shortages [1, 18–22]. Reportedly, 25%–55% of the nursing population in Australia and the United States (US) are 50 years and above [21, 23, 24]. Globally, the WHO has reported that, as of 2020, 17% of the 27.9 million nursing workforce is 55 years or older, indicating that they are expected to retire by 2030 [5]. Associated with the aging process, the growing prevalence of chronic diseases necessitates more preventative and complex surgical interventions, increasing the demand for perioperative nurses [25, 26].

The perioperative nursing sector faces recruitment challenges due to its historical background, regulatory frameworks across various nations, and distinctive characteristics. Despite being the first recognised nursing specialty in the late nineteenth century [27, 28], it has been suggested that perioperative nurses often struggle to assert their role, facing perceptions of being mere assistants to surgeons [29, 30]. Moreover, in some countries such as Spain and Turkey, perioperative nursing still lacks official recognition [31, 32]. Regulations in certain countries also pose barriers to entering the perioperative nursing specialty. While specialty nurses can be employed at different levels in Sweden and Greece [31] and nurses with experience in the intervention fields can practice in those areas in Portugal and Finland [31, 33], specialised postgraduate training is required for perioperative nurses before employment in countries such as Norway, Belgium, Lithuania, Netherlands, Poland, Slovenia, Germany, Switzerland, Brazil, China, Japan, and Canada [27, 31, 34–40]. In countries such as Spain and Turkey, highly centralised policy restricts the free-flowing labour market [31, 41]. The demanding nature of perioperative nursing may further deter potential recruits. Perioperative nursing is unique due to its rapid pace, involvement in complex procedures, reliance on advanced technical equipment, and quick patient turnover [4, 29, 42, 43]. Amidst the commitment to patient safety in a fast-paced and intricate setting [44, 45], perioperative nurses often face numerous hazards, including noise [46], chemicals, radiation, waste anaesthetic gases, disinfectants, surgical smoke, sharp objects, and bloodborne pathogens [44, 47, 48]. Moreover, stressors such as night shift demands, unforeseeable events, excessive workloads, and insufficient resources can lead to burnout among perioperative nurses [49]. The need for specialised skills and extensive orientation periods add to the complexity and costliness of training in perioperative nursing. Perioperative nurses require specialised critical care skills [50–52]. In addition to fundamental theoretical knowledge, they must possess advanced technical and cognitive abilities, such as anticipating patient needs, adapting to evolving situations, and efficiently handling unexpected events [53]. Therefore, it usually takes six to twelve months of orientation for new nurses to attain proficiency in their perioperative nursing roles [54, 55]. The extensiveness and length of training time make perioperative nursing one of the costliest specialties to train [22, 56]. The expense of training a perioperative nurse is believed to exceed that of general nursing, which can cost up to $88,000 per nurse [12, 57].

Within healthcare settings, nursing shortages can compromise the whole healthcare system. A shortage of nurses in a department leads to heightened workload and stress levels among the remaining nurses, leading to emotional exhaustion, burnout, and job dissatisfaction, ultimately impacting the quality of nursing care [7, 58, 59]. In the general nursing area, reviews have suggested a significant correlation between inadequate staffing levels and adverse patient outcomes [60, 61]. In the perioperative setting, research by Lee et al. [4] highlights a close relationship between nursing shortages and reduced quality of patient care. For example, an observational study carried out in postanaesthesia care units in Greece found that the incidence of hypoxaemia was significantly higher in understaffed units [62]. Nursing shortages can cause notable workforce instability, marked by higher turnover rates among nurses, leading to increased expenses associated with recruitment and training of replacements [7].

There has been abundant literature focusing on staffing shortages, primarily within the broader nursing field. A previous literature review by Ross [63] delved into factors contributing to nursing shortages and proposed solutions to mitigate them. Another systematic review pinpointed the following four primary contributors to nursing workforce shortages: policy and planning impediments, barriers to training and enrolment, causes of nursing staff turnover, and the stress and burnout experienced by nurses [58]. Regarding mitigating strategies to reduce nursing shortages, a systematic review by Park and Yu [64] evaluated the effectiveness of policies addressing nurse shortages. As examined in a systematic review by Mohamed and Al-Hmaimat [7], nurse residency programs emerged as promising educational interventions for nurturing competent new nurses and enhancing retention rates. Regarding the perioperative setting, a literature review by Willemsen-McBride [52] revisited preceptorship and suggested ways to enhance existing orientation programs. However, as most reviews focus on the general nursing landscape, there is a notable gap in exploring the staffing issues in the perioperative setting. Given the complexities involved in maintaining adequate staffing levels in perioperative environments, an integrative review was conducted. This study aimed to systematically explore the negative impacts, causal factors, and potential solutions related to shortages in perioperative nursing personnel. The Human Resource for Health and Action Framework was employed to structure and synthesise the information gathered. This framework, endorsed by the WHO, provided a robust framework for organising insights on human factors in healthcare [65, 66].

### 1.1. Objective

This integrative review aimed to explore contributors to the international perioperative nursing shortage. Strategies and initiatives to address perioperative nursing shortages and the potential impacts of shortages in this setting were also investigated.

### 1.2. Review Question

The main review questions are as follows:

What are the contributing factors and impacts of the perioperative nursing shortage worldwide? What strategies are used to address the shortages in the perioperative nursing workforce?

Using the Population, Prognostic Factors, and Outcome framework [67], the elements of the questions are framed as follows.

#### 1.2.1. Population

This study includes all perioperative nurses and undergraduate nursing students. Undergraduate nursing students were included to enhance understanding of how perioperative nurses are recruited into this specialty.

#### 1.2.2. Prognostic factors

Factors contributing to the perioperative nursing workforce shortage.

### 1.3. Outcomes of Interest

1. Factors contributing to workforce levels: recruitment, retention, and turnover.
2. Impacts of workforce shortages: at the patient level—quality of patient care and patient safety; at the organisational level—hospital revenue; and at the staff level—work and life quality, work satisfaction, health issues, and burnout.
3. Strategies to address workforce shortages.

## 2. Methods

This review used an integrative approach to assimilate both empirical and theoretical research [68–70]. This review followed the five steps of an integrative review: problem identification, literature search, data evaluation, data analysis, and presentation [71]. The search was reported according to the Preferred Reporting Items for Systematic Reviews and Meta-Analyses (PRISMA) statement [72].

### 2.1. Search Strategy

The search included the following databases: CINAHL, Embase, Emcare (via OVID), Medline (via EBSCOhost), Scopus, and Web of Science. Grey literature was sought via ProQuest Dissertations and Theses Global, Overton, and GreyNet. Key terms with MeSH headings included MH “perioperative nursing”, MH “operating room nursing”, MH “postanaesthesia nursing”, “surgical nurse”, “anaesthetic nurse”, (MH “recovery room”) AND (MH “nurses+”), “instrument nurse”, MH “personnel selection”, MH “personnel turnover”, “nurse recruitment”, “nurse retention”, “shortage of nurses”, “personnel staffing and scheduling+”, “nurse supply”, and “intention to leave”. Complete search strategies across all databases are provided in Supplementary File (available here) 1.

### 2.2. Inclusion and Exclusion Criteria

The search sought experimental and nonexperimental primary research studies of any design, policies, and reports published in English from January 2013 to June 2023. The inclusion criteria and excluding conditions for studies are outlined in Table 1.

### 2.3. Screening

The search process adhered to the flow diagram outlined in the PRISMA statement [72]. In the initial search phase, articles identified from targeted databases were exported to EndNote. Following this, duplicate entries were removed from the imported search results. The data were then forwarded to Rayyan for comprehensive screening, covering titles, abstracts, and full articles. Two independent reviewers conducted title and abstract screening according to predefined inclusion and exclusion criteria. In instances of disagreement, a third reviewer was consulted to reach a consensus. Full-text screening followed a similar procedure, with two independent reviewers assessing the articles and a third resolving any discrepancies. In this round, the identified articles underwent screening of both abstracts and full articles by two reviewers, with a third reviewer consulted to ensure consensus was reached.

### 2.4. Data Extraction

A data extraction form was devised to gather key elements and details from the chosen articles, which was piloted before implementation. This form encompassed categories such as “Author/Date/Country”, “Sample/Population”, “Study Method/Theoretical Framework”, “Contributing Factors and Negative Impacts of Shortages”, and “Strategies to Mitigate Shortages”. The primary reviewer extracted relevant data from the articles based on the predefined categories and entered it into the corresponding columns. The extraction process differed based on study type: themes identified from qualitative studies and reports were extracted, whereas descriptive data information was extracted from quantitative studies. The second reviewer examined the extracted data to confirm consensus.

### 2.5. Quality Appraisal

The Quality Appraisal for Diverse Studies (QuADS) tool was used to appraise the included 58 primary research articles due to its applicability for multi or mixed-methods design [73, 74]. The QuADS tool contains a checklist of 13 items with answers rated from 0 (lowest quality) to 3 (highest quality). Although the tool does not allow ranking the quality of studies as low, moderate, or high, the percentage of the maximum achievable score may indicate the quality of the appraised studies [73]. Two reviewers conducted appraisals independently, and disagreements were resolved through discussion. One reviewer was an author of an included article [75] and was purposefully not assigned to appraise this study.

Two viewers independently used the Quality Improvement Minimum Quality Criteria Set (QI-MQCS) [76] to evaluate the quality of the 26 quality improvement articles, with a third reviewer to resolve disagreements. Questions answered “met” scored “1” [76]. The total score of each article was added up, and a percentage based on a total mark of 16 was calculated.

### 2.6. Data Synthesis

**Figure 1:**
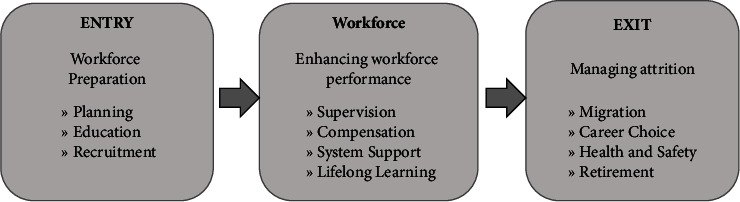
Human Resource for Health and Action Framework. Adapted from the human resource for health and action framework [65, 66].

The synthesis of data was meticulously structured and organised in alignment with the Human Resource for Health and Action Framework, a comprehensive model designed to foster the sustainability of the workforce by devising strategies to address critical workforce challenges such as inequitable staff distribution, insufficient skills and knowledge, elevated turnover rates, and diminished motivation [65, 66]. This framework serves as a guiding platform in navigating the complex terrain of workforce management within the perioperative nursing domain. The findings of the study were methodically condensed and categorised according to the three fundamental components of the framework: entry, current workforce, and exit, as illustrated in Figure 1. Each component represents a distinct phase in the lifecycle of the workforce, encompassing recruitment and onboarding processes, the ongoing management and development of the existing workforce, and the transition and departure of staff from the workforce [65, 66]. This systematic synthesis provides a structured approach to understanding the multifaceted challenges and opportunities within perioperative nursing workforce dynamics, thereby facilitating the development of effective interventions and strategies to address these complexities.

## 3. Results

**Figure 2:**
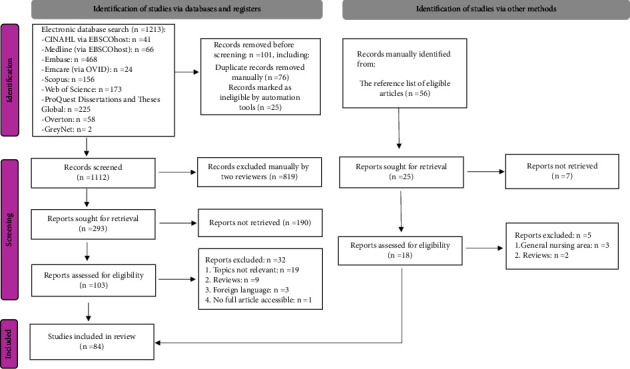
PRISMA flowchart [72].

This review encompassed 84 articles, as illustrated in Figure 2 of the PRISMA flowchart [72]. Most articles included in this review were conducted in the US (56/84, 67%). The remainder was conducted in Sweden (7/84, 8%), Australia (5/84, 6%), Canada (3/84, 4%), Iran (3/84, 4%), Turkey (2/84, 2%), and only one study (1% each) from the following: China, Finland, Ghana, Greece, Iceland, Norway, South Korea, and Spain. Most articles (58/84, 69%) comprise primary research using descriptive or observational designs, such as cross-sectional correlational surveys. The remaining 26 articles are quality improvement reports (26/84, 31%), primarily based in the US. The characteristics of the included 84 articles are included in the Study Characteristics Table (Supplementary File 2).

The critical appraisal results of the selected articles are presented in Supplementary File 3. The QuADS appraisal (part 1) reveals that 13 primary research articles (13/58, 22%) scored above 80% of the maximum score (39), 18 articles (18/58, 31%) scored between 60% and 79%, and the remaining 27 articles (27/58, 47%) scored below 60%. Appraisal of the 26 quality improvement reports using the QI-MQCS tool (part 2) resulted in 6 articles (6/26, 23%) scoring within 80–100% of the maximum score (16), 11 articles (11/26, 42%) scoring 60–79%, and 9 scoring below 60%. Although most of these reports scored higher than 60% (17/26, 65%), essential aspects of quality improvement reports such as study design, comparators, data source, adherence, and health outcomes are missed, which impacts the completeness of these reports [76, 77].

### 3.1. Shortage Levels

Limited information regarding the extent of shortages was observed in the selected articles. In the US, the Association of periOperative Registered Nurses (AORN) conducts annual surveys to examine compensation disparities, job satisfaction levels, potential turnover rates, and factors contributing to perioperative nurses' intentions to leave their positions [8, 78–85]. Notably, the ten AORN survey reports are the sole studies among the selected articles that provided data on vacancies. Over the ten years from 2013 to 2023, the surveys reported that the vacant percentage of full-time perioperative nursing positions rose from 3.1% to 18%, and the percentage of managers who claimed a moderate to crisis level of nursing shortages ranged from 37% to 73% [8, 78–85]. Conversely, we found a lack of data concerning perioperative nursing shortages in other countries.

### 3.2. Impacts of Perioperative Nursing Shortages

This review identified nursing shortages in the perioperative setting as an impact on the quality of patient care, nurse satisfaction, and health service revenue. Quality of patient care is generally affected by negative impacts on the remaining nurses due to understaffing, such as fatigue and burnout, which can lead to reduced job involvement and increased errors that harm patients [86]. A Turkish correlational study found that work-related stress may negatively impact safety attitudes by 20.2% [50]. An observational study of 2207 participants in Greece reported that the incidence of adverse events among patients was significantly higher and of higher severity in highly understaffed versus sufficiently staffed departments [62]. Another US survey covering 1693 perioperative nurses discovered that inadequate staffing in the perioperative settings caused missed care due to miscommunication and lack of preparation [87]. Some studies considered the financial impact of perioperative nursing shortages. In some hospitals, perioperative departments generate substantial revenue [88]. Financial loss is associated with delaying or cancelled surgeries due to a lack of nursing staff, as reported by the AORN surveys [8, 83–85], and the high turnover cost of US$65,0001–$120,000 for a perioperative nurse, according to a US quality improvement report [89].

### 3.3. Contributing Factors and Mitigating Strategies to Perioperative Nursing Shortages

In alignment with the Human Resources for Health and Action Framework [65, 66], a comprehensive examination was conducted to discern the factors contributing to and potential strategies to alleviate perioperative nursing shortages. This evaluation was structured around the framework's three pivotal phases: entry, current workforce, and exit. Each phase was meticulously scrutinised to identify primary themes indicative of the challenges and opportunities within perioperative nursing. The synthesis of these themes is presented in detail in Table 2, providing a comprehensive overview of the landscape surrounding perioperative nursing shortages and potential avenues for intervention.

#### 3.3.1. Entry (Workforce Preparation)

Workforce preparation encompasses strategic planning aligned with market needs, acquiring knowledge and skills through education and recruiting individuals into specialised roles within the field [65, 66]. This review revealed an imbalance between the demand for and the supply of perioperative nurses. The evidence indicates an increasing demand for perioperative nursing professionals. Almost 50% of the respondents to the AORN surveys in the US between 2013 and 2023 claimed increasing surgical activity in their facilities [8, 78–85]. Studies in the US [20, 85, 90, 91] and Spain [26] suggested that increasing demands for nurses are driven by an aging population requiring more frequent and intense surgical interventions. However, the availability of qualified perioperative nurses appears to be falling behind. The AORN surveys reported that only 3-4% of newly graduated nurses entered the perioperative setting annually [8, 78–85]. Likewise, additional studies included in the review found that the majority of nursing graduates opt for familiar nursing specialties, such as medical-surgical wards, instead of perioperative areas that were not generally offered during their undergraduate programs [12, 20, 22, 55, 56, 88–111].

The general practice of not recruiting newly graduated nurses into the perioperative specialty is another issue that further expands the gaps in meeting the demand. Particularly in the US, multiple quality improvement reports suggest a practice of not hiring new nursing graduates among perioperative nursing managers [43, 88, 92, 93, 105, 112–114]. Management's concern about a general lack of experience or understanding of perioperative nursing among graduate nurses was reported [43]. Authors of a quality improvement report identified that allowing novice nurses to develop critical thinking skills in medical-surgical units also decreases the probability of nurses transferring to perioperative areas once they are fully adapted to medical-surgical specialty [88]. This review highlighted various initiatives to alleviate staffing shortages, primarily concentrating on preparing and recruiting perioperative personnel. In the US, recruiting experienced nurses from other specialties and providing them with perioperative orientation was a frequently used strategy to fill vacancies in the perioperative section [8, 78–85, 93, 98, 101, 113, 115–117]. Furthermore, five reports highlighted that certain facilities enlist new nursing graduates into the specialty through on-the-job training initiatives, such as perioperative nurse residency programs [43, 92, 93, 112, 113]. Also, a trend for universities to embed perioperative nursing exposure programs for baccalaureate nursing students through health service-academic partnerships was evident [12, 20, 55, 56, 89–98, 100–112, 118]. One perioperative immersion program was designed for high school students [93].

Perioperative nursing programs successfully encouraged interest in entering the perioperative specialty and saved costs in orientation [22, 56, 88, 90, 91, 93–97, 100, 102–104]. It is reported that between 38%–100% of nursing students exposed to the perioperative setting entering the specialty after graduation [90, 94, 103, 105, 111, 113]. Specialty programs can help students make informed decisions about their career direction [106], with high retention rates (70%–100%) after two years recorded in four of the quality improvement reports [92, 93, 112, 119]. Studies from the US and Australia suggested that nursing students with perioperative exposure and guided learning can demonstrate practical skills in this specialty, with improved self-efficacy and confidence than those without [56, 88, 95, 96, 101]. The perioperative training and immersion programs also have benefits for hospital costs. Ten of the 26 quality improvement reports indicated that hospitals could save half of the costs in orienting a novice by employing newly graduated nurses with perioperative experience [56, 88, 93–96, 101, 103, 107].

#### 3.3.2. Current Workforce (Workforce Performance)

The articles examined in this study underscored that elevated nurse turnover significantly contributes to shortages within the perioperative nursing workforce. In the US, the AORN surveys reported that perioperative nursing turnover increased from 25% in 2016 to 59% in 2022; respondents who expressed intention to quit increased from 11% to 34% from 2013 to 2022 [8, 78–85]. Another study noted a consistent turnover of 12.8% to 13.6% between 2016 and 2017 [119]. In addition, a report indicated that approximately half of perioperative nurses exit the specialty within two to three years, often due to the extensive orientation requirements [88]. Intention to leave rates were reported as 20% in a Spanish study [26], 42.9% in an Iranian study [15], and deemed “high” in a South Korean study involving 193 perioperative nurses [120].

According to the Human Resource for Health and Action Framework, elevated turnover rates and leaving intentions are associated with supervision, compensation, system support, and learning opportunities within healthcare institutions [65, 66]. Constant supervision, such as implementing a routine monitoring process, can offer insights into the progress of key elements of human resource strategies [65, 120], but this beneficial practice has not been extensively adopted within healthcare systems. Besides the annual AORN survey [8, 78–85], other countries did not use similar benchmarking. Dissatisfaction with compensation is a contributing factor to perioperative nursing shortages. The ten AORN surveys identified that dissatisfaction with compensation increased from 17% to 52% from 2013 to 2022 [8, 78–85]. Salary was identified as a factor leading to the highest dissatisfaction among participants in two cross-sectional studies, one in Spain and one in the US [26, 121]. Dissatisfaction with compensation was also evident in two other studies [122, 123]. Lack of professional development opportunities can also lead to staff leaving their current positions, as reported by a focused ethnography of perioperative nurses in the US [124]. In Iceland, a cross-sectional study revealed that nurses provided opportunities for further training and education in another department or organisation were more likely to consider leaving their current position [125]. The foremost issue causing staff dissatisfaction, intentions to leave, and turnover is the inadequate organisational or managerial support to mitigate the risks of physical and mental health damage to the perioperative nurses. According to 23 articles in this review, organisations failing to provide a safe working environment or culture for perioperative nursing staff is a common issue leading to nurse turnover worldwide [8, 49, 75, 78–85, 118, 120, 122–134]. Potential factors posing physical and mental health damage to perioperative nurses in the workplace include environmental hazards such as biological, chemical, and radiation exposures [134], continuous physical exertion [82], and intense work demands requiring constant mental focus [120]. In addition, the persistence of negative behaviours such as incivility and bullying remains a significant challenge in the perioperative settings [20, 75, 122]. These behaviours encompass various forms, such as verbal abuse [120] and depersonalisation [26], along with instances of exclusion and mistreatment by colleagues and management [122, 132]. Lack of support or recognition from management also contributes to staff attrition [8, 80, 82–85, 118, 122, 123, 125, 128, 133]. Failing to provide flexible working hours also contributes to staff turnover. An average of 17.3% of AORN survey respondents reported dissatisfaction with working hours [8, 80, 82–85]. While a cross-sectional study in the US claimed that flexible scheduling was available to only 39% of 2121 perianaesthesia nurses [133], participants in another US qualitative study described a lack of proper breaks during long working hours [123]. Other issues that lead to nurse turnover associated with the system include budget constraints [8, 80–85], short of resources [75], lack of consistent guidelines and protocols for clinical practices [131], nursing staff not feeling connected to the hospital [118], and nurses being socially detached [120, 135].

Multiple strategies were identified to reduce turnover among the current workforce. Three Swedish descriptive studies [122, 126, 136] and two US-based phenomenological studies [118, 128] suggested that well-functioning leadership is vital to the success of other workforce strategies. Two studies recommended the importance of a healthy and safe work environment to job satisfaction and wellbeing among perioperative nursing staff [26, 75]. The significance of teamwork in the perioperative working environment was supported by a qualitative study in Norway [137], a correlational study in the US [138], and a focused ethnography in Canada [124]. Furthermore, an Iranian correlational study of 350 perioperative nurses identified a close association between professional communication and professional commitment [139]. A Swedish qualitative study identified that nursing staff who are provided with sufficient opportunities for career advancement have less intention to leave [136]. A high level of job satisfaction was reported in a cross-sectional study among perioperative nurses with higher certificate levels [121] and perioperative nurses who receive skill training [140]. In addition, the AORN surveys recommended the need for improvement in compensation [8,78–85]. Another correlational study stressed the importance of empowerment to staff retention [141]. Other suggested strategies to encourage retention include adequate staffing and resources [133], flexible hours, a supportive climate, and low-strain work to reduce health issues [135].

#### 3.3.3. Exit (Attrition)

Nurse attrition can be attributed to migration, retirement, health issues, or a change of career [65, 66]. This review found that mass retirement, health issues, and career changes lead to perioperative nursing attrition, but evidence of migration is not identified. Projected retirement among perioperative nurses is concerning. The retirement rate of perioperative nurses responding to the AORN surveys remained at an average rate of 34% between 2013 and 2023 [8, 80–85]. Another cross-sectional survey among 2121 perioperative nurses in the US found that a projected mass retirement (58%) was expected by 2020 [133]. A Spanish cross-sectional study identified that 20% of perioperative nurses will retire in 2024 [26, 118, 134, 142]. Health issues are also a critical factor for perioperative nurse attrition. Studies conducted in the US and Sweden identified that self-rated health and musculoskeletal disorders are associated with nurses leaving the profession [118, 126]. Change of career is another factor leading to nurse attrition. The ten AORN surveys identified perioperative nursing attrition due to new job opportunities or leaving the healthcare profession at a rate of 23.5% and 2%, respectively [8, 80–85]. A cross-sectional study in the US reported that nurses who can expand their skills and knowledge in other units or organisations are more likely to consider leaving their current position [125]. This review found limited evidence on strategies for managing attrition specifically. Participants in a phenomenological study targeting older nurses in the US reported fear of having to retire when their health deteriorates [118]. Strategies to help retention of older nurses include supportive leadership and health promotion programs to preserve their ability to remain on the job [118]. These strategies were also suggested in a Swedish qualitative study of 955 perioperative nurses [135].

#### 3.3.4. Overall Impression of Recommended Strategies

The strategies outlined in the chosen articles primarily focus on single dimensions, with a few incorporating multiple dimensions. Nonetheless, none of the studies employ comprehensive mitigation strategies, and there is a lack of reliable measurement tools to determine which strategies are more effective than the others. Although some articles in the review suggest coordinated recruitment and retention strategies to address current perioperative nursing shortages [8, 78–85, 90, 91, 93, 98, 101, 113, 115–117, 143], these strategies were not evident in the selected articles.

## 4. Discussion

Perioperative nursing shortages have been, and will continue to be, a challenge for health services over many years. According to a cross-sectional study in the US, the perioperative workplace was the least favourable among eleven nursing work environments [144]. This finding may be attributed to the fact that perioperative nurses face a greater risk of physical and mental injuries compared to general ward nurses, given the nature of their work environment and job responsibilities [118, 134, 142]. Perioperative nurses are often exposed to multiple physical and psychological stressors [45, 145]. This environment is known for its high demands, stress, and pressure [50, 51], requiring intense focus, expert knowledge, and swift action related to surgical procedures and patient safety [120]. In this high-risk environment, perioperative professionals frequently encounter the dilemma of ensuring patient safety amidst the rapid pace and intricate nature of modern surgical procedures [29, 146]. Multidisciplinary bullying and lateral violence persist, contributing to moral distress and potential mental health issues among perioperative nurses [29, 122]. The ongoing prevalence of burnout and mental exhaustion among perioperative nurses can significantly diminish their resilience and elevate their intentions to leave their positions [45, 122].

The review findings point out key factors affecting the perioperative workforce, such as rising demand for nurses, limited recruitment into the specialty, high turnover, and retirements. Significantly, while the increasing demands on nurses and the aging workforce are unpreventable, limited recruitment and high turnover emerge as factors that can be modified. Staffing preparation appears to lag due to a lack of undergraduate programs offering perioperative nursing training. Since its inception, perioperative nursing training initially adhered to a doctor-centric, hospital-based approach [29, 52]. However, in the 1980s, many hospital-based diploma nursing programs were either closed or shifted to baccalaureate degree programs, resulting in the omission of perioperative nursing from the curriculum [20]. Many universities are also discouraged from building up the curriculum as perioperative specialty not being a prerequisite for registration, lack of faculty with perioperative experience [90], time constraints, and shortages of local placements [29, 92]. Academic-practice teams may face obstacles when starting an immersion program, such as getting support from administrators and preceptors, coordinating schedules, managing time constraints, and dealing with limited staff and resources [91]. Consequently, nursing students typically graduate with minimal or no exposure to perioperative nursing [29]. Lack of immersion in the perioperative nursing role, primarily involving observation without hands-on experience, was linked to a decrease in the number of graduate nurses seeking positions in the perioperative specialty [55]. The high nurse turnover and elevated intention-to-leave rate among the perioperative nursing workforce are significant concerns. Factors contributing to perioperative nursing workforce turnover, as identified in this review, appear to be environmental, cultural, and managerial, highlighting implications for the wellbeing of perioperative nurses. It cannot be overstated that high-quality healthcare relies on healthcare practitioners' health, general wellbeing, and safety [147, 148]. Factors influencing the wellbeing of perioperative nurses include workplace culture, career development, work-life balance, and compensation [47, 142, 147]. However, a case study conducted in the UK declared that the wellbeing of the staff was either not a priority or not taken seriously by their employers [42]. The wellbeing crisis in health care has been identified as a key concern by many stakeholders [147–149]. The People Plan launched in both the US and the UK emphasised the importance of and proposed initiatives for looking after the health and wellbeing of healthcare employees [147, 150]. Psychological wellbeing is now a government priority in the workplace in Australia, with national standards and legislation established regarding workplace behaviours [151, 152]. A recent UK study with a three-round Delphi technique identified that actions to support the wellbeing of nurses should focus on the organisational level rather than public policy and research [147].

This review underscores the adverse effects of perioperative nursing shortages on existing staff, patient care quality, and organisational finances. These shortages contribute to staff fatigue and burnout, possibly lowering work engagement, escalating disruptive behaviours like incivility and bullying, and increasing the likelihood of errors jeopardising patient safety. In consequence, perioperative understaffing leads to further nursing turnover and subsequent financial loss in recruiting and training new staff [9]. Even the lowest rates (15–18%) of nursing workforce shortages disrupt hospital operations and increase hospital costs; a higher rate of nursing shortage may lead to substantial revenue loss and decreased quality of patient care for the healthcare system [10].

This review extensively explores strategies to alleviate the shortage of perioperative nurses. However, these strategies are mainly focused on the workforce preparation and recruitment phase. These strategies include offering perioperative nursing electives in undergraduate programs and orientation programs for newly graduated nurses or nurses from other specialties. While these training programs are beneficial for attracting and retaining new perioperative nurses, they may have drawbacks. The majority of articles advocating for educational programs are centred on the US, which limits the generalisability of this approach. In the US, nursing is the only healthcare profession with multiple entry-level educational pathways [153]. This concept may be applicable in countries such as the US, New Zealand, Australia, India, Sweden, and Greece, where specialty training can commence at various educational levels [29, 31, 93, 154–157]. However, it may not be the same in Portugal, where specialty skills can be acquired through localised training, and in countries such as Belgium, Poland, and the Netherlands, where postgraduate education is a prerequisite for entering the specialty [31]. In Spain, centralised workforce planning poses obstacles to specialisation programs for nurses, particularly as perioperative nursing remains unrecognised [31]. In Turkey, nursing holds a lower social status [158], and within perioperative nursing, the roles are limited to instrument and circulating nurses. Certification in this specialty requires two years of experience although specialised master's and doctoral programs are also offered [32].

Enhancing perioperative nursing recruitment via educational programs requires various factors to be considered. The success of training programs reportedly depends on the inclusion of all stakeholders, such as executive leaders, nursing managers, educators, preceptors, and students themselves, as suggested by two US-based quality improvement reports [22, 88]. Using a standardised course can help reduce local orientation cost and time, such as Periop 101: A Core Curriculum [43, 55, 56, 89, 101, 107, 112–114, 119], suggesting the need for similar programs globally. Facilities should weigh the drawbacks of solely focusing on recruiting and training new perioperative nurses. Many universities and local facilities lack the capacity to accommodate a surge in trainees [25]. AORN surveys indicate that experienced perioperative nurses often quit due to the strain of continuously training newcomers [8, 78–85]. Also, younger registered nurses were found to change employers faster than older nurses [25]. Reportedly, the turnover rate of perioperative nurses is between 13% and 75% in their first three years working in this specialty [88, 123, 159].

The retention of currently employed perioperative nurses is crucial, given the established link between the staffing of competent nurses and the quality of patient care. The knowledge and skills of senior nursing staff are a valuable resource in the perioperative department [29, 160]. As in other specialties, experienced perioperative nurses build up their knowledge, critical thinking, and extensive skills from years of experience and professional growth [160, 161]. The benefits of retaining these experienced perioperative nurses include saving costs in training new recruits [160], maintaining the stability and intellectual property of this nursing population, achieving high-quality patient care and having experienced mentors to guide students and novices [21, 150]. Strategies to manage perioperative nursing attrition, especially for nurses nearing retirement age, are understudied as per the selected articles. It has been indicated that perceptions about older perioperative nurses among nursing managers are often negative, according to qualitative studies conducted in Europe [162] and Australia [163]. Making this group of perioperative nurses active even postretirement could potentially help reduce the shortage levels. A scoping review in 2018 found that nurses' intentions to work after retirement ranged from 18.3% in Singapore to 73.2% in Australia [164]. The personal preference for delaying the retirement age, together with the intention of the government to raise the retirement age, for example, from 65 years to 67 years by 2028 in the UK, can be a promising solution for narrowing the nursing shortage gaps [165]. It was suggested that the retention of retirement-aged nurses be integrated into the national nursing workforce planning and workplace wellbeing policies [166].

This review highlights the importance and necessity of regular and routine monitoring of staffing issues within healthcare systems [65, 66]. Besides the ten AORN survey reports, which provided detailed insights into the contributing factors to shortages in the perioperative nursing workforce in the US, none of the other studies identified similar measures. Nonetheless, even with regular monitoring, the situation may vary in other countries. For instance, in Iceland, the turnover rate could be markedly different from that in the US, as nurses in certain specialties may not have the option to change workplaces without leaving their specialty or country despite having a high intention to leave [125]. In Spain, nurses are assigned according to healthcare authorities' planning and redistributive policies, which might drive nurses to explore job opportunities overseas, thereby exacerbating the nursing outflow [31, 168].

Programs such as the Magnet Recognition Program® can be used to help the retention of the current perioperative nurses. The Magnet Recognition Program®, established by the American Nurses Credentialing Centre in 1981, focuses on the establishment of professional organisational characteristics and achieving high job satisfaction for nurses [169]. Up to 2022, 591 hospitals in the US [170] and eight non-US hospitals hold Magnet® designation, three of which are in Australia [171]. Improved job satisfaction and reduced nursing turnover were reported by two Australian articles [172, 173]. Another cross-sectional study among Magnet®-employed nurses in Australia indicated a high level of job satisfaction and intention to stay in a better working environment than their international counterparts [171]. Internationally, multiple articles reported similar results [174–177]. A systematic review covering 17 papers supported the positive effect of Magnet® designation on the professional nurse practice environment, leading to reduced burnout, higher satisfaction, improved quality of care, and decreased intent to leave among Magnet® nurses [178]. This program, however, was not highlighted in the selected articles of the current review. It is suggested that health organisations consider this program or establish similar programs that fit local facilities.

## 5. Conclusion

This study examines the global shortage of perioperative nursing professionals, identifying key contributing factors such as high demand for surgical services, limited new entrants, and high turnover rates. While challenges like aging demographics are beyond control, interventions targeting workforce preparation and recruitment are proposed. However, limited evidence supports the retention of current perioperative nursing staff, with most focus on identifying contributing factors. Furthermore, the lack of comprehensive solutions and comparative assessments underscores the necessity for additional research. Extensive, practical, and adaptable approaches are crucial, particularly given the diverse regulatory landscapes across countries.

### 5.1. Implication for Nursing Management

The finding of this review highlighted the critical importance of perioperative nurses' wellbeing in relation to nursing retention. Increasing evidence suggests that effective leadership is critical to any multidimensional workforce planning strategy [21, 150]. As nursing managers shape the culture of a unit, perioperative nursing managers play a critical role in resolving staffing issues associated with workplace culture [11]. Any change initiatives, such as culture change, begin with management as the critical change agent [11, 179, 180]. They influence the professionalism of staff as role models [181]. The role of perioperative nursing managers in monitoring nursing turnover trends and evaluating the implications of workforce change has been emphasised, as less frequent monitoring may miss warning signs of workforce issues [167]. With support and resources from the organisation, nursing managers play an essential role in establishing a civil and collaborative work atmosphere and providing opportunities for staff development [148]. Based on a good understanding of human development as per Maslow's theory [182], managers providing career planning guidance to nursing staff according to their characteristics and level of experience is highly valued [183]. As effective leadership is critical to the success of the department, perioperative nursing succession planning should include perioperative nurse managers [161]. In addition, perioperative managers also have their own challenges [1, 2, 184, 185]. Besides “on-the-job training” opportunities [181], they should have continual access to various forms of support, ongoing education and training throughout their tenure as leaders [185], career planning, and continuous professional development so that they can support the workforce and address challenges [184]. The US has required specialty certification for perioperative managers as a standard of care to lead the transformative changes [181]. It is recommended that similar programs be used in other countries to allow perioperative leaders to acquire the knowledge and skills in their roles.

### 5.2. Indication for Further Research

Research on retaining older perioperative nurses as they approach retirement age is lacking. There is a need to understand their career plans, desired retirement age, and other factors affecting retirement decisions. In addition, while this review included 84 studies, the evidence varied in quality and utilised limited study designs. Furthermore, there is a lack of research or quality improvement programs employing coordinated methods and reliable measurement tools to address perioperative nursing shortages.

## Figures and Tables

**Table 1:** Eligibility criteria.

PFO	Included	Excluded
Population	(i) Instrument/circulating nurses	(i) Retired perioperative nurses (ii) Nurses working in other specialties
	(ii) Anaesthetic nurses/Nurse anaesthetists	
	(iii) Postanaesthetic care unit nurses	
	(iv) Perioperative nurses in management and education	
	(v) Nurse surgical assistants	
	(vi) Perioperative nurse practitioners	
	(vii) Perioperative ancillary nurses	
	(viii) Undergraduate nursing students involved in perioperative nursing programs	

Prognostic factors	Factors contributing to shortages in perioperative nursing	(i) Pandemic issues (ii) Natural disasters (e.g., fire) (iii) War, unstable political national environment (iv) Non work-related personal issues

Outcomes	(i) Workforce levels: recruitment, retention, turnover	(i) Critical condition of the patients (ii) Hospital revenue loss by other factors
	(ii) Impacts of workforce shortages: patient level-quality of nursing care and patient safety, organisational level-hospital revenue, staff level-burnout, work and life quality, job satisfaction, health issues, etc.	
	(iii) Strategies to address workforce shortages	

**Table 2:** Summary of contributing factors and strategies to address the perioperative nursing shortages.

Entry (Workforce Preparation)	

Planning	(−) An aging population leads to increasing demand for perioperative nurses
	(−) General lack of experienced/qualified perioperative nurses

Education	(−) No perioperative nursing programs for undergraduate nursing students
	(−) Lack of placement spaces in hospitals
	(−) Lack of perioperative nursing faculty in universities
	(+) Universities increasing faculty and providing perioperative nursing electives/programs for undergraduate students
	(+) Perioperative nursing educational programs for novices with health service: academic partnerships

Recruitment	(−) Not recruiting newly graduated nurses
	(+) Active hiring of newly graduated nurses
	(+) Recruiting and training experienced nurses from other specialty

Current Workforce (Enhance Workforce Performance)	

Supervision	(+) Yearly surveys to monitor job satisfaction and intention to leave (e.g., AORN surveys)

Compensation	(−) Insufficient salary/rewards for the roles and training, and compensation/salary topped out for senior nurses
	(+) Appropriate salary matching job roles, recruitment incentives, and retention bonus

Lifelong learning	(−) Lack of continuous training and chances for professional development
	(+) Continuous training opportunities
	(+) Having access to career advancement opportunities
	(+) Empowerment, autonomy, delegation of leadership, and governance
	(+) Career planning and customised training
	(+) Competitive working environment

System support	(−) Working environment with hazards that could lead to physical or psychological damage to staff
	(−) Various issues with management that lead to job dissatisfaction among staff
	(−) Inconsistent guidelines and protocols
	(−) Inadequacy of precautions
	(−) Facility budget constraints
	(−) Staffing staff feel no connection to the facility
	(+) Organisation level: stability, secured job, and resolving organisational issues
	(+) Increasing planned funding and providing adequate resources
	(+) Managers sufficiently trained in their roles, providing strong support, reducing hazards, and promoting staff wellbeing in the department

Exit (Managing Attrition)	

Migration	No evidence was found in the selected articles

Career choice	(−) New job opportunities in other organisations that could expand the nurses' skills and knowledge
	(−) Leaving the healthcare profession

Health and safety	(−) Musculoskeletal disorders due to aging or physical demands of the job
	(−) Poor workplace-related mental wellbeing
	(+) Developing practices or positions that require less physical exertion
	(+) Wellness programs focusing on health promotion and lifestyle, as well as reducing hazards
	(+) Readily accessible newer technologies to reduce physical strain (+) fewer call hours, shorter shifts, and flexible scheduling
	(+) Establish organisational policies, professional standards, and guidelines to prevent psychosocial hazards

Retirement	(−) Mass retirement due to an aging workforce
	(+) Supportive leadership and health promotion programs to improve job satisfaction and preserve the ability to remain on the job

*Note*: “(−)” indicates contributing factors to perioperative nursing shortages; “(+)” indicates strategies to resolve nursing shortages.

## Data Availability

Data sharing is not applicable to this article as no new data were created or analysed in this study.

## Additional Points

This review's strength lies in its rigorous and reliable study process, encompassing diverse data sources and study types and two reviewers' involvement in article selection and quality appraisal. However, restricting the language to English may have resulted in excluding relevant studies in other languages. In addition, the majority of studies was conducted in the US, potentially limiting the generalisability of findings to other countries.

## Supplementary Materials

Supplementary Materials The Supplementary Materials comprise three files detailing the search strategies, study characteristics, and appraisal results of the selected articles.
