# Supplementary material for: Perioperative Nursing Shortages: An Integrative Review of Their Impact, Causal Factors, and Mitigation Strategies
Source: J Nurs Manag. 2024 Aug 19;2024:2983251. doi: 10.1155/2024/2983251 (PMC11918777; doi:10.1155/2024/2983251)
Supplement: Supplementary Materials — The Supplementary Materials comprise three files detailing the search strategies, study characteristics, and appraisal results of the selected articles. [file 2983251.f1.pdf]

Supplementary File 1**:** Searching strategies for selected databases.

CINAHL (via EBSCOhost)

Limiters - Published Date: 20130101-20230601; Human; English

1. MH "Perioperative Nursing
2. MH "Operating Room Nursing"
3. MH "Postanesthesia Nursing"
4. “surgical nurse”
5. "anaesthetic nurse"
6. (MH "Recovery Room") AND (MH "Nurses+")
7. "instrument nurse"
8. S1 OR S2 OR S3 OR S4 OR S5 OR S6 OR S7
9. MH "Personnel Selection”
10. MH "Personnel Turnover"
11. “nurse recruitment”
12. “nurse retention”
13. “shortage of nurses”
14. MH "Personnel Staffing and Scheduling+"
15. “nurse supply”
16. “intention to leave”
17. S9 OR S10 OR S11 OR S12 OR S13 OR S14 OR S15 OR S16
18. S8 AND S17

Medline (EBSCOhost)

Limiters - Date of Publication: 20130101-20230601; Human; English

1. MH Perioperative Nursing+
2. MH Operating Room Nursing
3. MH Postanesthesia Nursing
4. “surgical nurse”
5. “anaesthetic nurse”
6. MH Recovery Room AND MH Nurses+
7. instrument nurse
8. S1 OR S2 OR S3 OR S4 OR S5 OR S6 OR S7
9. MH Personnel Selection
10. MH Personnel Turnover
11. nurse recruitment
12. nurse retention
13. shortage of nurses
14. MH Personnel Staffing and Scheduling+
15. nurse supply
16. intention to leave
17. S9 OR S10 OR S11 OR S12 OR S13 OR S14 OR S15 OR S16
18. S8 AND S17

Embase

Limiters - Date of Publication: 20130101-20230601; Human

1. perioperative AND nursing
2. operating AND room AND nursing
3. 'postanesthesia nursing'
4. anaesthetic AND nursing
5. surgical AND nursing
6. post AND anaesthetic AND recovery AND nursing
7. recovery AND room AND nuring
8. instrument AND nursing
9. scout AND nurse
10. scrub AND nurse
11. #1 OR #2 OR #3 OR #4 OR #5 OR #6 OR #7 OR #8 OR #9 OR #10
12. shortage
13. recruitment
14. retention
15. turnover
16. staffing AND issues
17. intention AND to AND leave
18. job AND satisfaction
19. #12 OR #13 OR #14 OR #15 OR #16 OR #17 OR #18
20. #11 AND #19
21. #20 AND [embase]/lim NOT ([embase]/lim AND [medline]/lim) AND ('article'/it OR 'conference abstract'/it OR 'review'/it) AND [humans]/lim AND [english]/lim AND [2013-2023]/py

Emcare (via OVID)

Limiters - Date of Publication: 2013-2023

1. perioperative nursing/
2. theatre nursing.mp.
3. operating room nursing/
4. postanesthesia nursing/
5. surgical nursing.mp.
6. scrub nurse.mp.
7. scout nurse.mp.
8. 1 or 2 or 3 or 4 or 5 or 6 or 7
9. shortage.mp.
10. retention.mp.
11. turnover.mp.
12. recruitment.mp.
13. staffing issues.mp.
14. intention to leave.mp.
15. job satisfaction/
16. 9 or 10 or 11 or 12 or 13 or 14 or 15
17. 8 and 16

Scopus

1. TITLE-ABS-KEY (perioperative AND nursing)
2. TITLE-ABS-KEY (operating AND room AND nursing)
3. TITLE-ABS-KEY (scout AND nurse)
4. TITLE-ABS-KEY (scrub AND nurse)
5. TITLE-ABS-KEY (recovery AND nurse)
6. TITLE-ABS-KEY (post-anaesthetic AND unit AND nurse)
7. TITLE-ABS-KEY (anaesthetic AND nurse)
8. TITLE-ABS-KEY (circulating AND nurse)
9. TITLE-ABS-KEY (ot AND nurse)
10. (TITLE-ABS-KEY (ot AND nurse)) OR (TITLE-ABS-KEY (circulating AND nurse)) OR (TITLE-ABS-KEY (anaesthetic AND nurse)) OR (TITLE-ABS-KEY (post-anaesthetic AND unit AND nurse)) OR (TITLE-ABS-KEY (recovery AND nurse)) OR (TITLE-ABS-KEY (scrub AND nurse)) OR (TITLE-ABS-KEY (scout AND nurse)) OR (TITLE-ABS-KEY (operating AND room AND nursing)) OR (TITLE-ABS-KEY (perioperative AND nursing))
11. TITLE-ABS-KEY (shortage)
12. TITLE-ABS-KEY (retention)
13. TITLE-ABS-KEY (recruitment)
14. TITLE-ABS-KEY (staffing AND issues)
15. TITLE-ABS-KEY (intention AND to AND leave)
16. (TITLE-ABS-KEY (shortage)) OR (TITLE-ABS-KEY (retention)) OR (TITLE-ABS-KEY (recruitment)) OR (TITLE-ABS-KEY (staffing AND issues)) OR (TITLE-ABS-KEY (intention AND to AND leave))
17. ((TITLE-ABS-KEY (ot AND nurse)) OR (TITLE-ABS-KEY (circulating AND nurse)) OR (TITLE-ABS-KEY (anaesthetic AND nurse)) OR (TITLE-ABS-KEY (post-anaesthetic AND unit AND nurse)) OR (TITLE-ABS-KEY (recovery AND nurse)) OR (TITLE-ABS-KEY (scrub AND nurse)) OR (TITLE-ABS-KEY (scout AND nurse)) OR (TITLE-ABS-KEY (operating AND room AND nursing)) OR (TITLE-ABS-KEY (perioperative AND nursing))) AND ((TITLE-ABS-KEY (shortage)) OR (TITLE-ABS-KEY (retention)) OR (TITLE-ABS-KEY (recruitment)) OR (TITLE-ABS-KEY (staffing AND issues)) OR (TITLE-ABS-KEY (intention AND to AND leave)))
18. ((TITLE-ABS-KEY (ot AND nurse)) OR (TITLE-ABS-KEY (circulating AND nurse)) OR (TITLE-ABS-KEY (anaesthetic AND nurse)) OR (TITLE-ABS-KEY (post-anaesthetic AND unit AND nurse)) OR (TITLE-ABS-KEY (recovery AND nurse)) OR (TITLE-ABS-KEY (scrub AND nurse)) OR (TITLE-ABS-KEY (scout AND nurse)) OR (TITLE-ABS-KEY (operating AND room AND nursing)) OR (TITLE-ABS-KEY (perioperative AND nursing))) AND ((TITLE-ABS-KEY (shortage)) OR (TITLE-ABS-KEY (retention)) OR (TITLE-ABS-KEY (recruitment)) OR (TITLE-ABS-KEY (staffing AND issues)) OR (TITLE-ABS-KEY (intention AND to AND leave))) AND (LIMIT-TO (PUBYEAR , 2023) OR LIMIT-TO (PUBYEAR , 2022) OR LIMIT-TO (PUBYEAR , 2021) OR LIMIT-TO (PUBYEAR , 2020) OR LIMIT-TO (PUBYEAR , 2019) OR LIMIT-TO (PUBYEAR , 2018) OR LIMIT-TO (PUBYEAR , 2017) OR LIMIT-TO (PUBYEAR , 2016) OR LIMIT-TO (PUBYEAR , 2015) OR LIMIT-TO (PUBYEAR , 2014) OR LIMIT-TO (PUBYEAR , 2013)) AND (LIMIT-TO (SUBJAREA , "NURS")) AND (LIMIT-TO (LANGUAGE , "English"))

Web of science

Timespan: 2013-01-01 to 2023-06-01 (Publication Date)

1. perioperative nursing (All field).
2. operating room nursing (All Fields)
3. surgical nursing (All Fields)
4. post anaesthetic nursing (All Fields)
5. post-operative nursing (All Fields)
6. circulating nurse (All Fields)
7. scout nurse (All Fields)
8. scrub nurse (All Fields)
9. anaesthetic nurse (All Fields)
10. OT nursing (All Fields)
11. #1 OR #2 OR #3 OR #4 OR #5 OR #6 OR #7 OR #8 OR #9 OR #10
12. shortage (All Fields)
13. staffing issues (All Fields)
14. retention (All Fields)
15. recruitment (All Fields)
16. work-life quality (All Fields)
17. job satisfaction
18. intention to leave (All Fields)
19. #12 OR #13 OR #14 OR #15 OR #16 OR #17 OR #18
20. #11 AND #19
21. #11 AND #36 and 1.14 Nursing (Citation Topics Meso) and English (Languages) and Nursing (Research Areas) and Nursing (Web of Science Categories).Timespan: 2013-01-01 to 2023-06-01 (Publication Date)

ProQuest Dissertations and Theses Global

1. (perioperative nursing)
2. (operating room nursing)
3. (operating theatre nursing)
4. (peri-anaesthesia nursing)
5. (post-anaesthetic nursing)
6. (anaesthetic nursing)
7. (scrub nurse)
8. (scout nurse)
9. (post-anaesthetic unit nurse)
10. (post-anaesthetic recovery nurse)
11. (recovery nurse)
12. (OT nurse)
13. (circulating nurse)
14. (perioperative nursing) OR (operating room nursing) OR (operating theatre nursing) OR (peri-anaesthesia nursing) OR (post-anaesthetic nursing) OR (anaesthetic nursing) OR (scrub

nurse) OR (scout nurse) OR (post-anaesthetic unit nurse) OR (post-anaesthetic recovery nurse) OR (recovery nurse) OR (OT nurse) OR (circulating nurse)
15. Shortage
16. Turnover
17. Staffing issues
18. Retention
19. Intention to leave
20. Personnel selection
21. Personnel recruitment
22. shortage OR turnover OR (staffing issues) OR retention OR (intention to leave) OR (personnel selection) OR (personnel recruitment)
23. ((perioperative nursing) OR (operating room nursing) OR (operating theatre nursing) OR (peri-anaesthesia nursing) OR (post-anaesthetic nursing) OR (anaesthetic nursing) OR (scrub nurse) OR (scout nurse) OR (post-anaesthetic unit nurse) OR (post-anaesthetic recovery nurse) OR (recovery nurse) OR (OT nurse) OR (circulating nurse)) AND (shortage OR turnover OR (staffing issues) OR retention OR (intention to leave) OR (personnel selection) OR (personnel recruitment)) AND (subt.exact("nursing") AND la.exact("ENG") AND diskw.exact("Job satisfaction" OR "Retention" OR "Turnover" OR "Nurse retention") AND pd(20130101-20230601))

OVERTON

((perioperative nursing) OR (operating room nursing) OR (operating theatre nursing) OR (peri-anaesthesia nursing) OR (post-anaesthetic nursing) OR (anaesthetic nursing) OR (scrub nurse) OR (scout nurse) OR (post-anaesthetic unit nurse) OR (post-anaesthetic recovery nurse) OR (recovery nurse) OR (OT nurse) OR (circulating nurse)) AND (shortage OR turnover OR (staffing issues) OR retention OR (intention to leave) OR (personnel selection) OR (personnel recruitment))

type Publication or Clinical guidance or Scholarly article or Periodical, published between 2013 and 2023, with topic 'Nursing' and with subject area 'labour employment employee'

GreyNet

Search through the website: https://easy.dans.knaw.nl/ui/datasets/id/easy-dataset:234556

Published datasets, search: nursing

Audience: interdisciplinary sciences

manual screening

Supplementary File 2: Study Characteristics Table

Author/Date /Country	Sample/ Population	Study Method /Theoretical framework	Contributing factors and negative impacts of shortages	Strategies to mitigate shortages
Ahanian et al., 2016; Iran	Perioperative nurses, n=226	Descriptive solidarity study; No theoretical framework noted	➢ Stress and burnout: Total stress in 94.6% of respondents is measured on average of 42.9% of respondents moderately tend to leave, and there is a significant relation between job stress and turnover intentions ($p<0.05$).	
Akgül & Aksoy, 2021; Turkey	Perioperative staff, n=164	Descriptive correlational research; No theoretical framework noted	➢ The mean total scale score of the Organisational Stress Scale was 3.28 ± 0.77, whereas the SAQ-OR (a scale measuring the attitudes of perioperative staff toward patient safety) mean total scale score was 54.27 ± 15.59. Regression analysis ($F= 42.250$; $P < .05$) showed that organisational stress had a statistically significant effect on attitudes toward patient safety ($t= -6.500$; $P < .05$). Organisational stress explained 20.2% of the change in patient safety attitudes (adjusted $R^2=0.202$).	
Arakelian & Rudolfsson, 2021; Sweden	Nurse managers, n=19	Qualitative; No theoretical framework noted	Challenges nursing managers face:<br>➢ Striving to treat employees with consideration and solicitude<br>➢ The obligation to take care of each employee's individual needs<br>➢ Convincing others was an uphill battle<br>➢ Finding solutions when things seem impossible<br>➢ Challenges in staff recruitment, allocation, and management<br>➢ Working with constantly changing planning<br>➢ Overwhelming amount of tasks<br>➢ Their own needs for caring and nurturing	➢ Careful selection of leaders<br>➢ Nurse managers are essential for job satisfaction and positive patient outcomes: willing to do good and care for one's employees, caring conversations,
Arakelian et al., 2019; Sweden	Nurse specialists, n=15	Qualitative; No theoretical framework noted	➢ Unwelcoming workplace<br>➢ One did not feel of value to the organisation<br>➢ Unsympathetic head nurse<br>➢ Colleagues with demeaning behaviour.	➢ A feeling of stability within the organisation and themselves.<br>➢ Good spirits between coworkers<br>➢ Feeling that everyone was of equal value in a good working team<br>➢ Caritative leadership: nursing managers/leaders being present, caring, role models, with humanity, being facilitators to professional development and wellbeing of the nurses<br>➢ Nursing staff have access to professional development
Arakelian et al., 2020; Sweden	Nurse managers, n=12	Qualitative; No theoretical framework noted		Leaders/managers:<br>➢ Being positive in their roles: Be the best self, be honest and fair, be strong for their employees, paving the way for them.<br>➢ Showing their employees respect and being a facilitator.<br>➢ Having continual access to various forms of support,<br>➢ With ongoing education and training throughout their tenure as leaders<br>➢ Having their 'secret room' to reflect on things and prepare oneself for difficult tasks

**Author/Date /Country**	**Sample/ Population**	**Study Method /Theoretical framework**	➢ **Contributing factors and negative impacts of shortages**	**Strategies to mitigate shortages**
Asimah Ackah & Adzo Kwashie, 2023; Ghana	Perioperative nurses, n=12	Qualitative; No theoretical framework noted	Staffing feeling stressed due to:<br>➢ The overwhelming demand that their job places on them<br>➢ Fears resulting from exposure to chemicals and body fluid<br>➢ Prolonged standing.<br>➢ Staff shortage, inadequate and faulty equipment, and inadequate supplies<br>➢ Interpersonal relationship-related problems such as lack of communication and disrespectful behaviours on the part of doctors contributed to stress<br>➢ Emotional issues related to death and dying<br>➢ Daily hassle (family responsibilities, financial difficulties, travel time to work, the long distances travelled to work)	Recommended strategies:<br>➢ Design better stress assessment programmes<br>➢ Employing more competent perioperative nurses<br>➢ Periodically provide workshops and in-service<br>➢ Provide training to ease stress resulting from nursing shortages and to reduce the nurses' work overload.<br>➢ Ensure adequate supply of equipment.<br>➢ Actively organising yearly workshops emphasising etiquette and attitude among surgical team members.<br>➢ Expand the infrastructure of the existing perioperative training facilities.
Bacon & Stewart, 2013, 2014, 2015, 2016, 2017, 2018, 2019, 2020, 2021, 2022; US	Perioperative nurses, n=ranged from 1895-2754	Cross-sectional; No theoretical framework noted	➢ Lack of qualified nurses (avg. 42%)<br>➢ Retirement (avg. 34%)<br>➢ Increasing surgical activity (avg. 50%)<br>➢ Budget/timing constraints (avg. 26%)<br>➢ Insufficient compensation (avg. 47%)<br>➢ Stress/burnout (psychological, avg. 35%);<br>➢ Undesirable workload, hours, or shifts (avg. 37%)<br>➢ Budget constraints (avg. 26%)<br>➢ High nursing turnover (avg. 41%), including poor working environment (avg. 44.2%), dissatisfaction with management (avg. 35%), dissatisfaction with employer (avg. 32.8%); personal reasons/family (avg. 9.4%), etc.	➢ Incentives<br>➢ Value and recognition<br>➢ Increased compensation
Ball et al., 2015; US	Nursing students, n=4	Report; No theoretical framework noted	➢ Lack of undergraduate exposure	Effective educational program for undergraduate nursing students, with positive effects:<br>➢ Increased interest in perioperative nursing led to two of the four senior nursing students being hired.<br>➢ Reduced the human resource costs of recruiting new staff<br>➢ Reduced orientation time and improved productivity for both the preceptor and the new nurse.
Beitz, 2019b; US	Perioperative leaders, n=27	Cross-sectional survey; No theoretical framework noted	➢ Mass retirement: Staff eligibility to retire in 3-5 years was 30%. Thirty-four percent (n=9) of leaders planned to retire in the next 3-5 years.<br>➢ Difficulty recruiting and retaining experienced perioperative nurses: Over two-thirds (n=21) reported this difficulty due to a lack of qualified, experienced personnel and poor work conditions (salaries, on-call requirements, etc.).<br>➢ Lack of newly graduated nurses with perioperative nursing experience, as claimed by perioperative directors (n=21)<br>➢ Lack of training opportunities: Over half (n=15) of directors had an on-the-job perioperative training program, and only two had formal training in local colleges.	➢ Effective educational programs

Author/Date /Country	Sample/ Population	Study Method /Theoretical framework	➢ Contributing factors and negative impacts of shortages	Strategies to mitigate shortages
Björn et al., 2015; Sweden	Perioperative nurses, n=147	Cross-sectional questionnaire; Attractive work model developed by Ateg and Hedlund		Bartlett's test found a 0.05 significance level for work conditions, work content, and job satisfaction contributing to job attractiveness.<br><br>➢ Work condition: relationship (13.5% variance), leadership (11.9% variance), equipment (9.6% variance), salary (8.7 % variance), organisation (8.4% variance), physical work environment (6.7% variance), location (5.8% of variance), working hours (5.5% of variance)<br>➢ Work content: mental work (24% of variance), autonomy (22.7% of variance), work rate (21.6% of variance)<br>➢ Job satisfaction: status (35.2% of variance), acknowledgement (25.8% of variance)
Björn et al., 2016; Sweden	Perioperative nurses, n=147	Correlational, cross-sectional survey; No theoretical framework noted	➢ Salary-a significant factor<br>➢ Not being able to influence their own work<br>➢ The physical work environment includes noise, air quality and the cleanness of the premises.<br>➢ Status: not having the opportunity to develop skills, not having positive challenges at work, not being able to do a good job, and not feeling competent.	The following had a statistically significant positive association with total attractiveness (32% of the variance):<br>➢ Work engagement ($p < 0.001$)<br>➢ Age ($p = 0.01$)<br><br>The following were rated highly in significance for work attractiveness:<br>➢ Relationships, teamwork and communication<br>➢ Leadership: transformational or relational leadership
Brinkman, 2013; US	New perioperative nurses, n=14	Ethnography; Leininger's Transcultural Care Theory	➢ Inconsistency in precepting<br>➢ Hostile and overwhelming environment<br>➢ Limited exposure before transition	Effective educational programs with characters as below:<br><br>➢ Positive learning experience<br>➢ Belonging and acceptance<br>➢ Stimulating environment<br>➢ Supportive personnel<br>➢ Consistency in precepting<br>➢ Collegiality among peers
Brooks et al., 2021; US	Nursing students, n=24	Report; No theoretical framework noted		➢ Effective educational programs: Now in its 3rd year, the program has a 100% retention rate among the cohorts. This improved retention rate netted a cost savings of $ 359,000. This program laid the foundation for new graduate nurses transitioning to a specialty, promoted new role formation, and strengthened the partnership and the profession.
Byrd et al., 2015; US	Graduate nurses, n=9	Report; No theoretical framework noted	➢ Lack of undergraduate exposure<br>➢ Lack of academic faculty<br>➢ Not recruiting new graduates	Effective educational program with characters as below:<br><br>➢ Simulation training sessions<br>➢ The use of a standardised course: Periop 101<br>➢ Being allowed to work on the modules from home.<br>➢ Acknowledgement and encouragement from experienced nurses and physicians

Author/Date /Country	Sample/ Population	Study Method /Theoretical framework	Contributing factors and negative impacts of shortages	Strategies to mitigate shortages
Ceschini, 2016; US	Undergraduate /graduate /novice nurses, n=125	Report; No theoretical framework noted	➢ Not recruiting new graduates	Effective educational programs with good results:<br><br>➢ Perioperative program for registered nurses without perioperative nursing experience: In the ten years, of the 125 graduate nurses and registered nurses without perioperative experience hired into the course, the facility has maintained an average retention rate of 85% for two years or more.<br>➢ Undergraduate nursing course: The recruited 15 registered students in one semester passed the course. Two of these 15 undergraduate nursing students were hired into the perioperative nursing program.<br>➢ Youth apprenticeship program for high school students: One high school student successfully completed this program with positive comments from perioperative staff.
Chipps et al., 2013; Australia	Periopeative nurses, surgical technologists, and unlicensed perioperative personnel, n=167	Descriptive correlational; A new model of bullying from Hutchinson et al.	➢ Stress: 62.2% considered their jobs either stressful or very stressful.<br>➢ Overtime working: 36.7% of respondents reported working more than their scheduled hours in the previous work week.<br>➢ Bullying acts: The intensity of bullying acts was 9.7 (SD 6.38). Respondents experienced an average of 2.1 (SD 3.75) acts weekly or daily (i.e., frequency)—approximately one-third (34%) of participants being the targets of workplace bullying.<br>The negative effect of bullying acts: Emotional exhaustion was moderate (mean 18.3 [SD 12.8]), with 47%, 30%, and 21.7% having low, moderate, and high emotional exhaustion, respectively. 3% of respondents indicated that bullying affected errors and perceptions of patient satisfaction, and 6% indicated that bullying affected patient safety.	➢ Job satisfaction: Concerning job satisfaction, 71.1% of respondents were either very satisfied or satisfied with their jobs.
Cox et al., 2023/ US	Nursing graduates, n=65 & 56	Descriptive survey research design with qualitative components; Schlossberg's transition framework		➢ Effective educational programs: 25 (38%) who participated in the perioperative elective entered the field after graduation. Furthermore, 7 (46.7%) of the 15 participants who had a precepted placement went on to work in perioperative nursing after graduation. Thirty-eight (68%) of 56 participants said they would consider a future job as a perioperative nurse.
Cramer et al., 2022; US	Perioperative nurses, n=13061	Cross-sectional design; No theoretical framework noted	Nurses were least satisfied:<br><br>➢ Nursing administration (range 3.80-4.05),<br>➢ Decision-making (range 3.33-3.86)<br>➢ Pay (range 3.33-3.88).	➢ Nurse-nurse interactions (top) (range 4.87-5.31).<br>➢ Task (range 4.33-4.75),<br>➢ Professional status (range 4.25-4.78),<br>➢ Nurse manager (range 4.24-4.71)<br>➢ Nurse-physician interactions (range 4.36-4.69)<br>➢ Professional development access (range 4.30-4.40),<br>➢ Career advancement opportunities (range 4.23-4.52)<br>➢ Autonomy (range 4.07-5.52),<br>➢ Job enjoyment (range 4.03-4.57)<br>➢ Higher certification status with higher job satisfaction

Author/Date /Country	Sample/ Population	Study Method /Theoretical framework	Contributing factors and negative impacts of shortages	Strategies to mitigate shortages
Doerner & Swenty, 2019; US	Undergraduate nurses, n=7	Report; No theoretical framework noted	➢ Lack of undergraduate exposure	➢ Effective educational programs: When completing the clinical immersion, the participating students perceived an increased growth in critical thinking, skill competency, and role socialisation and felt more equipped to function in the perioperative environment after graduation.
Eakin, 2015; US	Perioperative nurses and technicians, n=24	Phenomenological study; Herzberg's two-factor theory and Maslow's hierarchy of needs	➢ Work environment. Thirty-three percent of the participants indicated that working in an antagonistic and unfriendly working environment negatively influenced their job satisfaction.<br>➢ Recognition by surgeons: 4/24 participants expressed job dissatisfaction when they were not recognised by their leaders, surgeons, and anaesthetists for excelling in their jobs.<br>➢ Executive leadership: Thirty-three percent (33%) of the operating room registered nurses, and 50% of the surgical technologists expressed that they did not feel supported by hospital executive leaders in the operating room.	Job satisfaction influenced their intent to leave or remain in the operating room work environment. The themes include:<br><br>➢ Teamwork: 17/24 participants claimed experiences of teamwork to job satisfaction.<br>➢ Working with paediatric patients and their families: 11/24 participants expressed that working with paediatric patients and families increased their job satisfaction.<br>➢ Staff appreciation: Participants were more likely to stay at their jobs when they were appreciated by their peers, leaders, surgeons, and anaesthetists.
Eskola et al., 2016; Finland	Perioperative nurses, n= 96	Cross-sectional study; No theoretical framework noted	Operating room nurses agreed somewhat about their intention to leave their job (Md 4.7). Reasons:<br><br>➢ Overall job stress (M=2.3), with workload the highest (M=3.2);<br>➢ Low job satisfaction: issues of pay and prospects in their work (Md 3.4)<br>➢ Overall practice environment: nurses disagreed somewhat about their levels of empowerment (Md 2.8).	➢ The overall job satisfaction score (Md 4.5) suggested that they were somewhat satisfied with their work, and personal satisfaction was the highest reported construct of job satisfaction (Md 5.6).<br><br>➢ Overall practice environment: Operating room nurses judged the nurse–doctor relationship as the highest construct of their practice environment (Md 5.7).
Foran, 2015; Australia	Undergraduate nurses, n=332	Qualitative; No theoretical framework noted	Drawbacks of the educational programs:<br><br>➢ Instances of unintentionally non-guided experience<br>➢ The mentor was either uninterested or not supportive that there was insufficient proactive teaching or that tension between staff members prevented any teaching, and the students just stood and watched.<br>➢ Lack of patient contact<br>➢ They had to simply observe the lack of ability to be involved in the surgical procedure.	➢ Effective educational programs: Participants provided with a guided practical experience are more likely to consider perioperative nursing as a career, revealing that guided practice positively influences the recruitment of nurses to this specialty area.
Foran, 2016a; Australia	Undergraduate nurses, n=332	Quantitative; No theoretical framework noted	➢ Lack of undergraduate exposure	➢ Effective educational programs: scores of those who had been exposed to guided and non-guided practical experience. The t-test analysis revealed a p-value of < 0.001) and there is a statistically significant difference in the knowledge levels between the two groups (t ¼ 4.743; $p < 0.001$).

Author/Date /Country	Sample/ Population	Study Method /Theoretical framework	Contributing factors and negative impacts of shortages	Strategies to mitigate shortages
Foran, 2016b; Australia	Undergraduate nurses, n=161 & 172	Quantitative and qualitative; No theoretical framework noted	➢ Lack of undergraduate exposure: 18.3% of participants did not set foot in an operating suite in their entire undergraduate education, and a further 9.7% had five hours or less	➢ Effective educational programs: Ninety per cent of guided participants enjoyed their placement in theatre and considered future employment in perioperative nursing.
Fujihara & FitzGerald, 2020; US	Undergraduate nursing students	Report; No theoretical framework noted	Challenges of the educational program:<br>➢ Developing and conducting the program was labour-intensive and costly.<br>➢ Demands on preceptors seemed out of proportion with the small number of students.<br>➢ Difficulty ensuring support for students during the recently graduated nurse residency program.<br>➢ Integrating student nurses into surgical teams that traditionally do not have novice nurses was challenging.	➢ Effective educational programs: The summer perioperative nursing practicum enhances collaboration between academic-service partners to address nursing shortages in highly specialised areas of nursing practice
Goliat et al., 2021; US	Undergraduate nursing students and novices	Report; No theoretical framework noted	Recruiting nurses in the Perioperative Services Departments can also be challenging due to the following issues:<br>➢ The high volume of looming retirements<br>➢ Student nurses have little or no exposure to perioperative nursing.<br>➢ Academic-practice teams may encounter potential barriers in launching an immersion, such as lack of administrative and preceptor buy-in, difficulties in coordinating academic-practice schedules, insufficient time, and lack of staff and resources.	➢ Effective educational program: Academic-practice partnerships and the development of immersions, however, have important implications for heightening levels of student engagement, fostering residency-ready nurse graduates, sustaining specialty areas of nursing, and fulfilling practice needs.
Gorgone et al., 2016; US	Newly graduated RNs, n=12	Report; No theoretical framework noted	➢ Not recruiting new graduates	➢ Effective educational program: The two cohorts of 6 graduate nurses who completed the program have been hired. A retention rate of 100% was attained. In one year, the facility recovered the initial program costs.
Gregory et al., 2014; US	Undergraduate nursing students and novices	Report; No theoretical framework noted	➢ Lack of undergraduate exposure	➢ Effective educational programs: This PACU internship promoted teamwork, collaboration, and nurse satisfaction. As a result, the overall turnover rate for the PACU slowly declined from 15.9% in 2008 to 9.2% in 2011. In 2010 and 2011, our participation by PACU nurses in the nurse satisfaction survey was 100%.
Gu et al., 2021; South Korea	Perioperative nurses, n=193	Cross-sectional; No theoretical framework noted	➢ Aging workforce<br>➢ Poor working environment<br>➢ Low job satisfaction<br>➢ Stress/burnout<br>➢ Lack of social support	

Author/Date /Country	Sample/ Population	Study Method /Theoretical framework	Contributing factors and negative impacts of shortages	Strategies to mitigate shortages
Hall, 2021; US	Perioperative nurses, n=55	Quantitative; Kanter's theory of structural empowerment		Overall, the sample's intent to stay is above average: Mean 4.09 (2-6), SD=1.14.<br>➢ Structural empowerment: moderate level of empowerment, Mean=19.23 (11-29), SD=4.40, highest in the access to opportunity (Mean=3.95) and lowest in organisational relationships subscale (Mean=3.05)<br>➢ Improved job satisfaction: above average job satisfaction, Mean=3.74 (2-5), SD=0.71 and scored highest in the intrinsic subscale (M=3.84). A statistically significant predictor of intent to stay as per regression analysis.
Heinzelman, 2013; US	Baby Boomer Perioperative nurses, n=10	Phenomenological interview study; No theoretical framework noted	Attrition of baby boomers:<br>➢ Do not feel connected to the hospital<br>➢ Deterioration of health was the main reason for the retirement of baby boomers	➢ Relationships with patients and performing the daily duties of nurses are a source of satisfaction for baby boomer nurses.<br>➢ Fairness, support/concern, and delegation skills are nurse leader qualities that might shape the meaning perioperative nurses attach to remaining in their jobs.<br>➢ Various executive leadership qualities may improve job satisfaction and retention in the perioperative setting.
Helzer Doroh & Monahan, 2016; US	Undergraduate nursing students	Report; No theoretical framework noted	➢ Lack of undergraduate exposure	➢ Effective educational programs: Thus far, the perioperative preceptorship program at Oakland University has succeeded, as indicated by the number of students who have secured employment in the perioperative department and the number of clinical sites seeking student placements.
Holmes et al., 2020; Norway	Perioperative nurses, n=10	Qualitative; No theoretical framework noted		➢ Team skills/teamwork: Good team skills impact the performance of perioperative nursing and patient safety and can contribute to a better work environment.
Jay, 2015; US	Perioperative nurses, n=322	Correlational study; Social rule systems theory	Turnover score, mean=36.3 (SD=13.6), range:14-81<br>➢ Aging workforce<br>➢ Poor working culture: poor teamwork, lack of autonomy	➢ Workplace environment: the mean Physician-Nurse Collaboration score =26.1 (SD = 2. 0), range: 19-28; with a weak negative correlation between anticipated Turnover and Collaboration: $r_s = -0.12$; $p = 0.03$.<br>➢ The mean Nurse Autonomy score =15.5 (SD = 0.9), range: 11-16); Weak negative correlation between Turnover and Autonomy: $r_s = -0.13$; $p = 0.01$<br>➢ Personal positive attitude

Author/Date /Country	Sample/ Population	Study Method /Theoretical framework	Contributing factors and negative impacts of shortages	Strategies to mitigate shortages
Kapaale, 2020; US	Student nurses, n=38	Quantitative; Theory of Planned Behaviour	➢ Lack of undergraduate exposure	➢ Personal attitude<br>➢ Subjective norm (e.g. affirmation by nurse managers)<br>➢ Perceived Behavioural control (e.g. the availability of positions, preparation/training, and competitive rewards)<br><br>The above components explain 64.62% of the variance influencing the intention to choose perioperative nursing. All subscales correlated highly ($r \geqslant 0.5$) with the intention subscale.
Kiekkas et al., 2019; Greece	Post-anaesthesia care unit nurses, n=2,207	Observational study; No theoretical framework noted	Negative impacts of understaffing:<br><br>➢ Episodes of hypoxia: sufficient staffing: 7.1%, severity: 4.5 (2.0-6.7); low understaffing: 10.5%, severity 5.5 (3.0-8.5); high understaffing: 15.1%, severity: 6.0 (4.0–9.0)<br>➢ Episodes of hypotension: sufficient staffing: 4.5%, severity: 11.0 (4.0-18.0); low understaffing: 9.4%, severity 18.0 (12.0-25.0); high understaffing: 10.1%, severity: 19 (13.0–29.0)<br>➢ Episodes of bradycardia: sufficient staffing: 6.1%, severity: 8.0 (5.0-12.0); low understaffing: 7.7%, severity 7.0 (5.0-10.0); high understaffing: 6.7%, severity: 7.5 (5.0–11.0)	
Krefetz, 2015; US	Perioperative nurses, n=101	Observational study; No theoretical framework noted		➢ Continuous training: Time management training was associated with a significant ($p < .05$) increase in the test scores for jobs, job satisfaction, and opportunity for promotion satisfaction, with substantial effect sizes reflecting practical significance.
Kwak, 2020; US	Nurse anaesthetists, n= 299	Exploratory, descriptive design; No theoretical framework noted	➢ Poor working environment impacting job satisfaction: 89.8% reported experiencing or witnessing workplace incivility at least yearly. Only 10.2% reported never experiencing or witnessing incivility. Over 50% of responses reflect experiencing or witnessing incivility at least once a week, and over a third of the respondents employed at a teaching facility reported experiencing or witnessing incivility at least once a day.	
Laflamme et al., 2019; Canada	Perioperative nurses, n=11	Focused ethnography/ secondary analysis; Framework in interprofessional relations		➢ Interprofessional relations<br>➢ Recognition at work<br>➢ Nurses' character traits (Resilience)

Author/Date /Country	Sample/ Population	Study Method /Theoretical framework	Contributing factors and negative impacts of shortages	Strategies to mitigate shortages
Lang et al., 2022; Australia	Perioperative nurses, N=239	Correlational study; Hutchinson's model of bullying in the nursing workplace	➢ Poor working environment: over half of perioperative nurses (n = 158/257, 61%) were exposed to workplace bullying. Consequences included fatigue and exhaustion (n = 129/192, 67%), anxiety (n = 123/192, 64%) and sleeplessness (n = 121/192, 63%). Organisational processes ($r = .458$, $p < .001$), bullying acts ($r = .289$, $p < .001$) and avoidance and withdrawal at work ($r = .440$, $p = .001$) increased burnout. Psychosocial distress ($r = .216$, $p < .001$) was associated with decreased resilience)	➢ Shared values regarding professional behavioural expectations<br>Ongoing improvements to an organisational climate of psychosocial safety
Lee et al., 2020; Canada	Acute-care nurses (including perioperative specialty), n=113	Secondary analysis of cross-sectional survey; No theoretical framework noted	19% of the variance in perioperative RNs' intention to leave their jobs in the next year.<br><br>➢ High workload: job satisfaction β (95% CI)=−0.06 (−0.14, −0.08); intention to leave: odds ratio (95% CI)=1.18 (0.76, 1.84).<br>➢ Emotional exhaustion: job satisfaction: $\beta = -0.21$, $p < .001$; intention to leave: Odds ratio (95% CI)= 1.75 (1.25, 2.11).	➢ Leadership and support of nurses: β (95% CI)= 0.16 (−0.01, 0.35)<br>➢ Staffing adequacy: β (95% CI)=0.07 (−0.16, 0.31)<br>➢ Collegial nurse-physician relationship: β (95% CI)=0.21 (0.04, 0.49)
Lögde et al., 2018; Sweden	Perioperative specialist nurses, n=20	Qualitative individual in-depth interviews; No theoretical framework noted	Themes of reasons why specialist nurses quit their jobs:<br><br>➢ The head nurses' betrayal and dismissive attitude, and not feeling needed (main reason);<br>➢ Colleagues' diminishing behaviour (main reason);<br>➢ Inhumane working conditions leading to the adverse health effects;<br>➢ Not being free to decide about one's life and family life is more important than work;	
Lunsford, 2023; US	Perioperative nurses, n= 34	Qualitative; Roy's adaptation model	Five major themes that caused fatigue, stress, dissatisfaction, or burnout in the perioperative nurses:<br><br>➢ High patient-to-nurse ratios<br>➢ Understaffing<br>➢ Lack of appreciation or respect<br>➢ Inconsistent guidelines and protocols<br>➢ Increasing expectations, pressure, and bullying	
Manchester et al., 2019; US	Novice perioperative nurses	Report; No theoretical framework noted		➢ Effective educational programs: The pilot program demonstrated positive outcomes, and in response, a systematic evaluation with future cohorts is being developed.
Manchester et al., 2023; US	Undergraduate nursing students, n=27	Report; No theoretical framework noted	➢ Lack of undergraduate exposure	➢ Effective educational programs: The program provides collaborative learning to train new perioperative nurses with successful outcomes regarding participant satisfaction, self-perceived readiness for practice, and facility cost savings.

**Author/Date /Country**	**Sample/ Population**	**Study Method /Theoretical framework**	**Contributing factors and negative impacts of shortages**	**Strategies to mitigate shortages**
Manz et al., 2021; US	Undergraduate nursing students	Report; No theoretical framework noted	➢ Lack of undergraduate exposure	➢ Effective educational programs: The program generated interest in the perioperative nursing specialty, resulting in four students being hired into the partnering healthcare system as perioperative nurses. The student evaluation of the program has highlighted the need for the addition of perioperative education to the undergraduate curriculum, a thorough perioperative orientation for students new to the perioperative setting, and sufficient training for perioperative preceptors.
Marsh et al., 2020; US	Perioperative nurses, n= 1,693	Cross-sectional survey; Kalisch's missed nursing care model	➢ Poor communication and preparation: overall, respondents' perceptions of the frequency of missed nursing care were low (0.84 on a scale of 0 to 4 [0 = never, 4 = always]), including communication (43.8%), suspension of activities during the surgical time out (39.9%), implementation of isolation precautions (36.1%), use of a standardised hand-over communication tool (33.7%), reporting of abnormal laboratory results (33.4%).	➢ Sufficient staffing: Adequate staffing causes less missed care than inadequate staffing ($B = -0.062$, $SE = 0.011$, $P < .001$)<br>➢ Higher level of education: Nurses with associate degrees and nurses who graduated from diploma programs reported less missed care than nurses with higher degrees ($B = -0.147$, $SE = 0.0336$, $P = .001$).
Mattioni & Wilson, 2018; US	Undergraduate nursing students	Report; No theoretical framework noted	➢ Lack of undergraduate exposure	➢ Effective educational program: 75 students have participated in the program. Of these 75 students, 41 (approximately 55%) have applied to open positions, and 40 (approximately 53%) have been hired. Higher percentages of applicants and hires were observed at program inception because more positions were open then. Of the 53% of students who were hired, there has been a 100% retention rate after two years of employment. This is significantly higher than the two-year retention rate of 39% for new-to-practice nurses reported in the literature.
Mayes & Cochran, 2023/ US	Perioperative nurses, n= 26	Qualitative; No theoretical framework noted	Factors that negatively influence decisions to resign, such as:<br>➢ Negative leader characteristics (e.g. favouritism)<br>➢ Negative social interactions (e.g., incivility)<br>➢ Unplanned shift extensions (e.g., overtime)<br>➢ Safety concerns<br>➢ Diverging goals (e.g., patient care, economics)	Interventions to create a work environment that encourages wellbeing, including:<br>➢ Support for work-life balance,<br>➢ A culture of respect and teamwork<br>➢ Competitive compensation packages<br>➢ Leaders should monitor interventions for effectiveness. Hiring perioperative leaders with perioperative experience
Miandoab et al., 2016; Iran	Perioperative nurses, n=71	Qualitative; No theoretical framework noted		Job involvement increases when:<br>➢ The work is suitable for the abilities, attitudes, and other features of employees;<br>➢ Have personnel interested in the job, positive spirit, and self-confidence;<br>➢ Establishing a work atmosphere that will enable positive work experience for nursing staff.

Author/Date /Country	Sample/ Population	Study Method /Theoretical framework	Contributing factors and negative impacts of shortages	Strategies to mitigate shortages
Monahan, 2015; US	Undergraduate nursing students and novices	Report; No theoretical framework noted	➢ Lack of undergraduate exposure	➢ Effective educational program: As hoped, this collaborative effort between a healthcare institution in need of perioperative nurses and a university has addressed the nursing shortage for these hospitals. By providing a supportive environment, the student nurses have been successful in obtaining the skills needed to provide patients with a positive surgical experience. It has also provided the student nurses with a 'real' perioperative experience, while permitting the institution an opportunity to be certain that the student is a 'fit' within the perioperative department thus addressing the retention issues and concerns of these healthcare institutions
Moultrie, 2023; US	Perioperative nurses, n=12	Qualitative research of two surveys; Framework in predicting nurse retention	➢ Not recruiting new graduates	➢ Effective educational program: The results of this study suggested that opportunities are needed to strengthen and improve perioperative nurse residents' learning and work environment as they transition into professional practice.
Nash et al., 2018; US	Undergraduate nursing students, n=8	Report; No theoretical framework noted	➢ Lack of undergraduate exposure	Effective educational program: Seven of the eight students reported considering pursuing a career in perioperative nursing after the internship. Four of the students responded the perioperative department would be their first choice.
Nissen, 2020; US	Nursing graduates, n=3	Report; No theoretical framework noted	➢ Not recruiting new graduates	➢ Effective educational program: After the first cohort graduated, facility leaders and surgeons were receptive to continuing the program. The perioperative nurse resident expressed high satisfaction with the hospital-sponsored residency program, and the enthusiasm of these recently hired perioperative nurses has infused renewed energy into our current perioperative workforce.
Parnikh et al., 2022; Iran	Perioperative nurses, n=350	Correlational design, census; No theoretical framework noted		➢ Professional communication: mean scores of 136.81 ± 13.77, highest in the dimension of interprofessional interactions (mean 32.96 ± 5.66)<br>➢ Professional commitment: mean score of 71.42 ± 11.62, highest in the dimension of involvement in one's profession (mean=37.46 ± 4.81); Correlation the operating room nurses' professional communication and professional commitment mean scores ($p < 0.001$, $r = 0.235$)<br>➢ Female<br>➢ Married status<br>➢ Interest in work<br><br>The relationship between the participants' professional commitment mean scores on the one hand and their gender ($p = 0.041$), marital status ($p = 0.047$), and interest in work ($p = 0.000$) on the other was found to be statistically significant.

Author/Date /Country	Sample/ Population	Study Method /Theoretical framework	Contributing factors and negative impacts of shortages	Strategies to mitigate shortages
Penprase et al., 2016; US	undergraduate nursing students and graduates, n=18	Report; No theoretical framework noted	➢ Not recruiting new graduates<br>➢ Lack of undergraduate exposure	➢ Effective educational program: To date, the program has 18 student graduates in two semesters; all have been offered positions in the perioperative setting, and 14 have accepted positions.
Pfander & Breznau, 2018; US	Novice perioperative nurses	Report; No theoretical framework noted		➢ Effective educational program: Implementing a peri-anaesthesia nurse residency program improved the department's vacancy rate while maintaining the quality of care expected at a Magnet organisation.
Reinhart et al., 2021; US	Novice perioperative nurses	Report; No theoretical framework noted		➢ Effective educational program: The mean cardiovascular procedures knowledge questionnaire preintervention score was 63%, and the mean postintervention score was 80%. The cardiovascular procedures Periop 202 program may provide healthcare systems with an evidence-based tool to recruit and train nurses interested in cardiovascular procedures nursing . It will allow these nurses to develop their confidence in a new specialty, thereby assisting with nurse retention. '
Reuter & King, 2021; US	Undergraduate nursing students	Qualitative; No theoretical framework noted	➢ Lack of undergraduate exposure	➢ Effective educational program: Recently graduated nurses with perioperative educational experience have a better understanding of the role of a perioperative nurse. Increasing prelicensure student exposure to the perioperative clinical environment is associated with positive outcomes for the department, organisation, nurses, and patients. Similar results in other specialties may be possible.
Reuter & King, 2023; US	Undergraduate nursing students, n=40	Report; No theoretical framework noted	➢ Lack of undergraduate exposure	➢ Effective educational program: Students reported significant gains in knowledge, critical thinking, teamwork, and confidence at course completion, but the post-test mean number of students interested in pursuing perioperative nursing was lower than the pretest mean. This realisation is perceived as a positive outcome of the perioperative elective course because it may decrease turnover among newly hired perioperative nurses.
Reyka, 2015; US	Perioperative nurses n=45	Cross-sectional; Lazanis' Stress theory	➢ Poor working environment: disruptive behaviour: Mean=38.33 (22.0-55.0), low job satisfaction: Mean=62.97 (49.0-76.0), intent to leave: Mean=8.55 (3.00-12.0), stress: Mean=24.46 (12.0-40.0), coping: Mean=40.37 (19.0-60.0). A strong negative correlation exists between disruptive behaviour and satisfaction (-0.67). A moderate to strong positive correlation existed between disruptive behaviour and intent to leave (0.46).	

Author/Date /Country	Sample/ Population	Study Method /Theoretical framework	Contributing factors and negative impacts of shortages	Strategies to mitigate shortages
Rogers et al., 2020; US	Undergraduate nursing students, n=34	Report; No theoretical framework noted	➢ Lack of undergraduate exposure	➢ Effective educational program: During the first three years of the partnership, 34 students completed the course. Nine students accepted perioperative nurse externships, and nine graduates accepted perioperative nurse positions. All the students who completed the perioperative nursing course passed the NCLEX-RN examination on the first attempt.
Ross, 2017; US	Peri-anaesthesia nurses, n=2,121	Cross-sectional; No theoretical framework noted	➢ Flexible scheduling: available to only 39% of the paranesthesia nurses<br>➢ Manager responsibility for multiple units: most nurses (53%) responded that their manager was responsible for three or more units,<br>➢ Projected mass retirement: 81% of the participants in this study are Baby Boomers, and 58% of the nurses responding reported that they will retire by 2020.	➢ Assigned preceptor<br>➢ Education status: The mean score for manager support differed significantly (*p* =0 .04) between nurses with a master's degree (M 3.83, standard deviation [SD] 5 .82) and nurses with a professional education diploma (M 3.58, SD 5 .84). Certified nurses have more positive perceptions of workload (*p* =0 .00) and intent to stay (*p*=0.00) than noncertified nurses.
Ruth-Sahd & Wilson, 2013; US	Undergraduate nursing students, n=6	Report; No theoretical framework noted	➢ Lack of undergraduate exposure	➢ Effective educational program: By offering this course, academic institutions respond to the changing and demanding perioperative workforce. By taking this course, students may confirm if this is the specialty area they want to work on graduation. The perioperative education staff and perioperative nurses view this as a win-win situation because it allows them to showcase their specialty area and embrace new nurses. Nursing faculty members also have similar beliefs as they feel they are meeting the needs of their local hospital in which students complete much of their clinical requirements. Faculty members also believe that offering this course meets the needs of their community of interest.
Saxton & Nauser, 2020; US	Undergraduate nursing students, n=6	Qualitative; Kolb's theory	➢ Lack of undergraduate exposure	➢ Effective educational program: Of the six students, five indicated that their experiences in the course affirmed their intent to pursue employment in that setting following graduation.
Schmidt & Brown, 2019; US	Undergraduate nursing students, n=23	Cross-sectional survey; No theoretical framework noted	➢ Lack of undergraduate exposure	➢ Effective educational programs: 83% responded that they would consider pursuing a position in perioperative nursing.
Sherman, et al., 2014; US	Perioperative nurse leaders, n=256	Qualitative; No theoretical framework noted	➢ Aging workforce<br>➢ General lack of young, qualified perioperative nurses and leaders	➢ Set up a strategic plan for the future perioperative nursing workforce

Author/Date /Country	Sample/ Population	Study Method /Theoretical framework	Contributing factors and negative impacts of shortages	Strategies to mitigate shortages
Sillero-Sillero & Zabalegui, 2020; Spain	Perioperative nurses, n=130	Cross-sectional questionnaire; No theoretical framework noted	In general, 20% of perioperative nurses would want to quit. The least favourable characteristic of the work environment:<br>➢ Skill, leadership and support of those responsible (M = 2.27, SD= 0.67; range 2.15–2.39)<br>➢ Suitability of personnel and the resources: M = 2.12, SD = 0.65; range 2.01–2.23)<br>➢ Participation of nurses in hospital affairs (M =1.96, SD = 0.43; range 1.88-2.03, including participating in hospital decisions (M = 1.33, SD = 0.62), an administration that listens and responds to employee concerns (M=1.56, SD = 0.73) and opportunities to ascend within the organisation (M =1.65, SD = 0.70)	Favourable characteristics present in the work environment:<br>➢ Nursing fundamentals for quality (M = 2.65, SD=0.52; range 2.56–2.74, including working with clinically competent nurses (M = 3.51, SD = 0.65), care are based on a nursing model (M = 3.16, SD = 0.91) and administrators expect a high level of care (M = 2.89, SD = 1.18)<br>➢ Nurse-physician relationships (M = 2.47 SD = 0.68; range 2.35–2.59).
Sveinsdóttir & Blöndal, 2014; Iceland	Surgical nurses, n=189	Cross-sectional survey design; No theoretical framework noted	Variables with a positive association with intention to leave:<br>➢ Lower general job satisfaction [odds ratio (OR) = 0.393]<br>➢ Not being praised by the head nurse (OR = 0.523)<br>➢ Not working in a competitive work climate (OR = 0.542)	➢ Expanding opportunities (OR = 1.250),<br>➢ Having one's knowledge respected (OR = 1.738)<br>➢ Not engaging in unprofessional work (OR = 1.909)
Tschirch et al., 2017; US	Undergraduate nursing students	Report; No theoretical framework noted	➢ Lack of undergraduate exposure	➢ Effective educational program: Using the perioperative practice area to provide a foundational clinical rotation equips students with the fundamental skills they will implement throughout their nursing careers and introduces them to this specialised practice in a way that few other programs offer.
Uğurlu et al., 2015; Turkey	Perioperative nurses and technicians, n=74	Qualitative questionnaire; No theoretical framework noted	➢ Perioperative nurses and technicians are exposed to significant risk factors.<br>➢ Hospital precautions are insufficient to protect workers from these risk factors.<br>➢ Some participants are not aware of risk factors;<br>➢ Reported stress levels of perioperative personnel are high, and the most common causes of occupational stress are insufficient communication and teamwork.	
Vortman et al., 2019; US	Novice perioperative nurses, n=27	Report; No theoretical framework noted		➢ Effective educational program: Offering perioperative nursing experiences for undergraduate nursing students may serve as a practical approach to improve recruitment, retention, and the overall nursing shortage in specialty areas and, depending on the program design, this may generate cost savings for organisations.
Wålinder et al., 2018; Sweden	Perioperative nurses, n=955	Cross-sectional questionnaire; No theoretical framework noted	One-third also had thought of leaving health care during at least one month the previous year:<br>➢ Lower social support<br>➢ High demands, together with low control	Descriptive results show that most reported good or high work ability (96%) and moderate to high zest for work (76%).<br>➢ High social support<br>➢ Low strain

Author/Date /Country	Sample/ Population	Study Method /Theoretical framework	Contributing factors and negative impacts of shortages	Strategies to mitigate shortages
Yan et al., 2021; China	Perioperative nurses, n=1,418	Cross-sectional survey; No theoretical framework noted		Self-career planning of perioperative nurses impacted by:<br><br>➢ Perioperative nurses with higher organisational career management perception (β = 0.753, p <.001) and those working at tertiary hospitals (β = 0.042, p =.022) pay more attention to self-career planning. Monthly incomes (β = −0.563, p =.045) could negatively predict the intention of nurses to conduct a self-career planning.<br>➢ Access to career advancement: Implementation of organisational career management<br>➢ Other factors are age, length of service, professional title, education level, employment method, and marital status.
Zekveld & Berquist, 2022; Canada	Undergraduate nursing students, n=13	Report; No theoretical framework noted	Students identified the following concerns:<br><br>➢ Poor communication and collaboration between the preceptors at the hospital sites and the faculty;<br>➢ Lack of preparedness among students;<br>➢ Challenges with perioperative staff members, including a lack of support for the program from the hospital site staff members;<br>➢ Heavy workload with the AORN Periop 101 course, as this was assigned on top of the general course workload required of all fourth-year students.	➢ Effective educational program: Of the 13 students who participated in the perioperative consolidation program, 9 (69%) completed the survey. When asked about their intention of seeking a job in the perioperative setting after placement, all 9 (100%) of the respondents indicated that they intended to seek employment in the perioperative specialty. All but one (17%) of the leadership team members who responded to the survey offered their placement students a job in the perioperative setting.

Supplementary File 3: Appraisal results

**Part 1: Appraisal results for the selected primary studies (n=58) using the Quality Appraisal for Diverse Studies (QuADS) tool (Harrison et al., 2021).**															
**Authors**	**Q1**	**Q2**	**Q3**	**Q4**	**Q5**	**Q6**	**Q7**	**Q8**	**Q9**	**Q10**	**Q11**	**Q12**	**Q13**	**Total Score**	**Percentage (out of full mark-39)**
Akgul & Aksoy, 2021	1	3	3	3	1	3	2	2	1	2	3	0	1	25	**64%**
Ahanian et al., 2016	1	2	2	1	1	1	1	1	1	0	1	0	0	12	**31%**
Arakelian & Rudolfsson, 2021	1	2	2	2	2	1	1	2	1	2	3	0	2	21	**54%**
Arakelian et al. 2019	1	3	3	3	2	1	1	1	2	3	2	1	3	26	**67%**
Arakelian et al., 2020	1	2	2	3	2	0	1	1	1	2	3	1	2	21	**54%**
Asimah Ackah & Adzo Kwashie, 2023	3	3	3	3	3	2	2	2	2	3	3	1	2	32	**82%**
Bacon & Stewart, 2013	1	1	3	1	2	0	0	1	1	2	3	0	0	15	**38%**
Bacon & Stewart, 2014	1	1	3	2	1	0	0	1	1	2	2	0	0	14	**36%**
Bacon & Stewart, 2015	1	1	1	2	1	0	0	1	1	2	2	0	0	12	**31%**
Bacon & Stewart, 2016	1	1	1	2	1	0	0	1	1	2	2	0	0	12	**31%**
Bacon & Stewart, 2017	1	1	3	2	1	0	0	1	1	1	2	0	0	13	**33%**
Bacon & Stewart, 2018	1	1	1	2	1	0	0	1	1	2	2	0	0	12	**31%**
Bacon & Stewart, 2019	1	1	1	2	1	0	0	1	1	1	2	0	0	11	**28%**
Bacon & Stewart, 2020	1	1	1	2	1	0	0	1	1	2	2	0	0	12	**31%**
Bacon & Stewart, 2021	1	1	1	2	1	0	0	1	1	2	2	0	0	12	**31%**
Bacon & Stewart, 2022	1	1	1	2	1	0	0	1	1	2	2	0	0	12	**31%**
Beitz, 2019b	2	3	2	2	1	1	1	1	2	1	1	1	1	19	**49%**
Björn et al., 2015	2	3	3	3	3	3	3	3	2	3	3	1	2	34	**87%**
Björn, et al., 2016	2	3	3	3	2	3	3	2	3	3	3	1	1	32	**82%**
Brinkman, 2013	3	3	3	3	3	2	3	3	3	3	3	1	1	34	**87%**
Chipps et al., 2013	3	3	2	3	1	3	3	3	3	2	3	1	3	33	**85%**
Cox et al., 2023	2	3	2	2	2	2	3	2	3	2	3	0	3	29	**74%**
Cramer et al., 2022	1	3	2	2	3	3	3	2	3	3	3	0	2	30	**77%**
Eakin, 2015	3	3	3	3	3	2	2	3	3	3	3	2	3	36	**92%**
Eskola et al., 2016	3	2	3	2	2	3	3	2	2	2	3	1	2	30	**77%**
Foran, 2015	2	1	2	1	1	1	1	2	2	2	3	1	2	21	**54%**
Foran, 2016a	3	2	2	2	2	3	3	2	1	2	2	2	0	26	**67%**
Foran, 2016b	1	2	2	2	1	2	2	1	0	1	2	1	0	17	**44%**
Gu et al., 2022	1	3	2	3	2	3	3	2	2	2	3	1	1	28	**72%**

Hall, 2021	3	3	3	3	3	3	3	3	3	2	3	1	3	36	**92%**
Heinzelman, 2013	3	3	3	3	3	2	2	3	1	3	3	2	2	33	**85%**
Holmes et al., 2020	3	3	3	2	3	3	2	2	2	2	2	1	2	30	**77%**
Jay, 2015	3	3	3	3	1	3	3	3	3	3	3	2	3	36	**92%**
Kapaale, 2020	3	3	3	3	1	3	3	3	1	3	3	2	2	33	**85%**
Kiekkas et al., 2019	1	3	3	2	1	2	2	2	3	3	3	0	3	28	**72%**
Krefetz, 2015	2	3	2	1	2	3	2	2	2	2	2	1	3	27	**69%**
Kwak, 2020	3	3	2	2	2	3	3	3	3	3	3	2	3	35	**90%**
Laflamme et al., 2019	2	1	2	2	1	2	0	1	1	2	3	0	1	18	**46%**
Lang et al., 2022	3	3	3	3	2	2	3	3	2	1	3	0	3	31	**79%**
Lee et al., 2020	1	2	3	3	2	3	3	2	2	2	3	2	2	30	**77%**
Lögde et al., 2018	1	3	2	2	2	2	2	2	2	3	3	1	2	27	**69%**
Lunsford, 2023	1	3	1	1	1	1	0	1	0	2	1	0	1	13	**33%**
Marsh et al., 2020	2	3	3	2	3	3	2	2	2	3	3	2	2	32	**82%**
Mayes & Cochran, 2023	1	3	2	3	1	2	2	2	2	3	3	0	2	26	**67%**
Miandoab et al., 2016	1	1	1	0	0	0	0	1	0	1	1	0	0	6	**15%**
Moultrie, 2023	3	3	2	2	1	3	2	1	1	3	3	2	3	29	**74%**
Parnikh et al., 2022	1	1	2	2	1	2	2	1	1	2	2	0	1	18	**46%**
Reuter & King, 2021	1	1	3	1	0	1	0	1	0	1	1	1	1	12	**31%**
Reyka, 2015	3	3	3	3	3	3	3	3	3	3	3	2	2	37	**95%**
Ross, 2017	1	3	2	2	1	2	1	1	2	2	2	0	1	20	**51%**
Saxton & Nauser, 2020	3	3	3	2	2	1	2	3	0	2	3	0	2	26	**67%**
Schmidt & Brown, 2019	1	1	2	1	1	1	0	1	1	1	0	0	1	11	**28%**
Sherman et al., 2014	1	2	2	1	1	0	0	1	1	1	1	0	1	12	**31%**
Sillero-Sillero & Zabalegui, 2020	1	2	2	2	1	2	3	1	1	1	1	0	1	18	**46%**
Sveinsdottir & Blondal, 2014	2	2	2	2	1	2	3	1	1	2	2	2	3	25	**64%**
Uğurlu, et al., 2015	1	2	3	2	1	2	3	1	1	2	1	1	0	20	**51%**
Wãlinder et al., 2018	2	3	3	2	1	3	3	1	1	3	3	0	1	26	**67%**
Yan et al., 2021	1	2	1	1	1	2	2	1	2	2	1	1	1	18	**46%**
**80-100% n=13 (22%)**<br>**60-79% n=18 (31%)**<br>**<60% n=27 (47%)**			Q1: Theoretical or conceptual underpinning to the research; Q2: Statement of research aim/s; Q3: Clear description of research setting and target population; Q4: The study design is appropriate to address the stated research aim/s; Q5: Appropriate sampling to address the research aim/s; Q6: Rationale for choice of data collection tool/s; Q7: The format and content of data collection tool is appropriate to address the stated research aim/s; Q8: Description of data collection procedure; Q9: Recruitment data provided; Q10: Justification for analytic method selected; Q11: The method of analysis was appropriate to answer the research aim/s; Q12: Evidence that the research stakeholders have been considered in research design or conduct; Q13: Strengths and limitations critically discussed (Harrison et al., 2021).											

Part 2: Appraisal results for quality improvement reports (n=26) using the Quality Improvement Minimum Quality Criteria Set (QI-MQCS) tool (Hempel et al., 2015)																		
**Article**	**Q1**	**Q2**	**Q3**	**Q4**	**Q5**	**Q6**	**Q7**	**Q8**	**Q9**	**Q10**	**Q11**	**Q12**	**Q13**	**Q14**	**Q15**	**Q16**	**Score out of 16**	**Percentage**
Ball et al., 2015	met	met	met	met	met	met	met	met	met	met	met	met	met	met	met	met	16	**100%**
Brooks et al., 2021	met	met	met	met	met	met	met	met	met	not met	met	met	not met	met	not met	not met	12	**75%**
Byrd et al., 2015	met	met	met	met	met	met	met	not met	not met	not met	not met	not met	not met	met	not met	met	9	**56%**
Ceschini, 2016	met	met	met	met	met	met	not met	met	not met	not met	not met	not met	met	met	met	not met	10	**63%**
Doerner & Swenty, 2019	met	met	met	met	met	met	met	met	met	not met	not met	met	met	met	met	met	14	**88%**
Helzer Doroh & Monahan, 2016	met	met	met	met	met	not met	not met	not met	not met	not met	not met	met	met	met	not met	not met	8	**50%**
Fujihara & FitzGerald, 2020	met	met	met	met	met	met	met	not met	met	not met	not met	met	met	met	met	not met	12	**75%**
Goliat et al., 2021	met	met	met	met	met	met	not met	met	met	not met	not met	met	met	met	met	not met	12	**75%**
Gorgone et al., 2016	met	met	met	met	met	not met	not met	not met	met	met	not met	met	not met	met	met	not met	10	**63%**
Gregory et al., 2014	met	met	met	met	met	met	met	not met	met	not met	not met	met	met	met	met	not met	12	**75%**
Manchester et al., 2019	met	met	met	met	met	met	not met	not met	met	not met	not met	met	not met	met	met	met	11	**69%**
Manchester et al., 2023	met	met	met	met	met	met	met	met	met	not met	not met	met	met	met	met	not met	13	**81%**
Manz et al., 2021	met	met	met	met	met	met	met	met	met	met	not met	met	met	met	met	met	15	**94%**
Mattioni & Wilson, 2018	met	met	met	met	met	met	not met	met	met	met	met	not met	met	not met	not met	not met	11	**69%**
Monahan, 2015	met	met	met	not met	met	not met	not met	not met	met	not met	not met	not met	not met	not met	met	not met	6	**38%**
Nash et al., 2018	met	met	met	met	met	not met	met	met	met	met	not met	not met	not met	not met	met	not met	10	**63%**
Nissen, 2020	met	met	met	met	met	not met	not met	not met	not met	not met	not met	met	met	not met	met	not met	8	**50%**

Penprase et al., 2016	met	met	met	met	met	not met	not met	not met	met	not met	not met	not met	met	met	met	not met	9	**56%**
Pfander & Breznau, 2018	met	met	met	met	met	met	met	not met	met	not met	not met	not met	not met	not met	not met	not met	9	**56%**
Reinhart et al., 2021	met	met	met	met	met	met	met	met	met	not met	not met	met	not met	not met	not met	not met	10	**63%**
Reuter & King, 2023	met	met	met	met	met	met	met	met	met	met	not met	met	met	met	met	met	15	**94%**
Rogers et al., 2020	met	met	met	met	met	not met	not met	met	met	not met	not met	not met	met	met	not met	not met	9	**56%**
Ruth-Sahd & Wilson, 2013	met	met	met	met	met	not met	not met	not met	not met	not met	not met	met	not met	met	met	not met	8	**50%**
Tschirch et al., 2017	met	met	met	met	met	not met	not met	met	met	not met	not met	met	not met	not met	not met	not met	8	**50%**
Vortman et al., 2019	met	met	met	met	met	met	met	not met	met	not met	not met	not met	not met	met	met	met	11	**69%**
Zekveld & Berquist, 2022	met	met	met	met	met	met	met	met	met	not met	not met	met	met	met	met	not met	13	**81%**

80-100% n=6 (23%)
60-79% n=11 (42%)
<60 % n=9 (35%)

Q1. Organisational Motivation: Organisational problem, reason, or motivation for the intervention; Q2. Intervention Rationale: Rationale linking the intervention to its expected effects; Q3. Intervention Description: Change in organisational or provider behaviours; Q4. Organisational Characteristics: Demographics or basic characteristics of the organisation; Q5. Implementation: Temporary activities used to introduce potentially enduring changes; Q6. Study Design: Study design and comparator; Q7. Comparator: Information about comparator care processes; Q8. Data Source: Data source and outcome definition; Q9. Timing: Timing of intervention and evaluation; Q10. Adherence / Fidelity: Adherence to the intervention; Q11. Health Outcomes: Patient health-related outcomes; Q12. Organisational Readiness: Barriers and facilitators to readiness; Q13. Penetration / Reach: Penetration / reach of the intervention; Q14. Sustainability: Sustainability of the intervention; Q15. Spread: Ability to be spread or replicated; Q16. Limitations: Interpretation of the evaluation (Hempel et al., 2015).
